# A survey of isatin hybrids and their biological properties

**DOI:** 10.1007/s11030-024-10883-z

**Published:** 2024-06-04

**Authors:** Vanessa Asoh Shu, Donatus Bekindaka Eni, Fidele Ntie-Kang

**Affiliations:** 1 Center for Drug Discovery, Faculty of Science, University of Buea, Buea, Cameroon; 2 Department of Chemistry, Faculty of Science, University of Buea, Buea, Cameroon; 3 Institute of Pharmacy, Martin-Luther University Halle-Wittenberg, Halle (Saale), Germany

**Keywords:** Isatin hybrids, Biological properties, Pharmacophore, Molecular hybridization, Structure–activity relationship

## Abstract

The emergence of diverse infections worldwide, which is a serious global threat to human existence, necessitates the urgent development of novel therapeutic candidates that can combat these diseases with efficacy. Molecular hybridization has been established as an efficient technique in designing bioactive molecules capable of fighting infections. Isatin, a core nucleus of an array of compounds with diverse biological properties can be modified at different positions leading to the creation of novel drug targets, is an active area of medicinal chemistry. This review containing published articles from 2005 to 2022 highlights isatin hybrids which have been synthesized and reported in the literature alongside a discussion on their biological properties. The enriched structure–activity relationship studies discussed provides insights for the rational design of novel isatin hybrids with tailored biological properties as effective therapeutic candidates inspired by nature.

**Supplementary Information:**

The online version contains supplementary material available at 10.1007/s11030-024-10883-z.

## Introduction

**Fig. 1:**
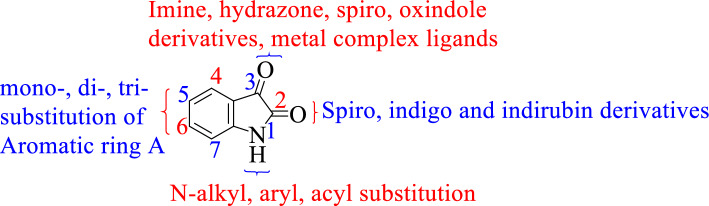
The various possible modification positions on the isatin scaffold [12]

Isatin **1** (indol-2,3-dione: Fig. 1), a secondary metabolite of tryptophan, has been found to be widely distributed in the central nervous system, mammalian tissues, and body fluids of humans [1–3]. This oxidized indole has been used as the core structure in the formulation of several compounds which have been tested and identified as potent inhibitors of Apoptosis [4–9], Anticonvulsants[10, 11], antiviral [5, 12–16], antitubercular [17–19], antifungal [20, 21], antimicrobial [22, 23], antioxidant [24, 25], Antimalarial [26, 27], and Anti-inflammatory agents [28, 29]. Among the known heterocyclic compounds, quinoline and its derivatives have been used for the development of novel drug entities, thus gaining significant attention among 21st-century scientists [30]. Triazole hybrid compounds and aminoquinoline and derivatives have shown promise in the development of next generation antimalarials [31, 32]. Xanthone conjugated amino acids, *N*-methylpicolinamides, and dihydrazones were recently shown to portray anticancer activities against a broad array of cancer cell lines [33–35], while dihydrazone analogs have shown promise as potential antibacterials [36]. Isatin is also considered a versatile and favorable precursor for pharmacophore development as a privileged scaffold [9] because the moiety can be modified at various positions (N-1, C‐3, C‐4, C‐5, and C‐7 positions) as illustrated in Fig. 1, resulting in different derivatives with diverse biological properties [37, 38]. The modifications at the N‐1, C‐3, and C‐5 positions are much more favorable with the mono-substitution at the C-5 position considered the most favorable. The C-5 position is beneficial to control the electronic effect, lipophilicity, and physicochemical properties.

**Fig. 2:**
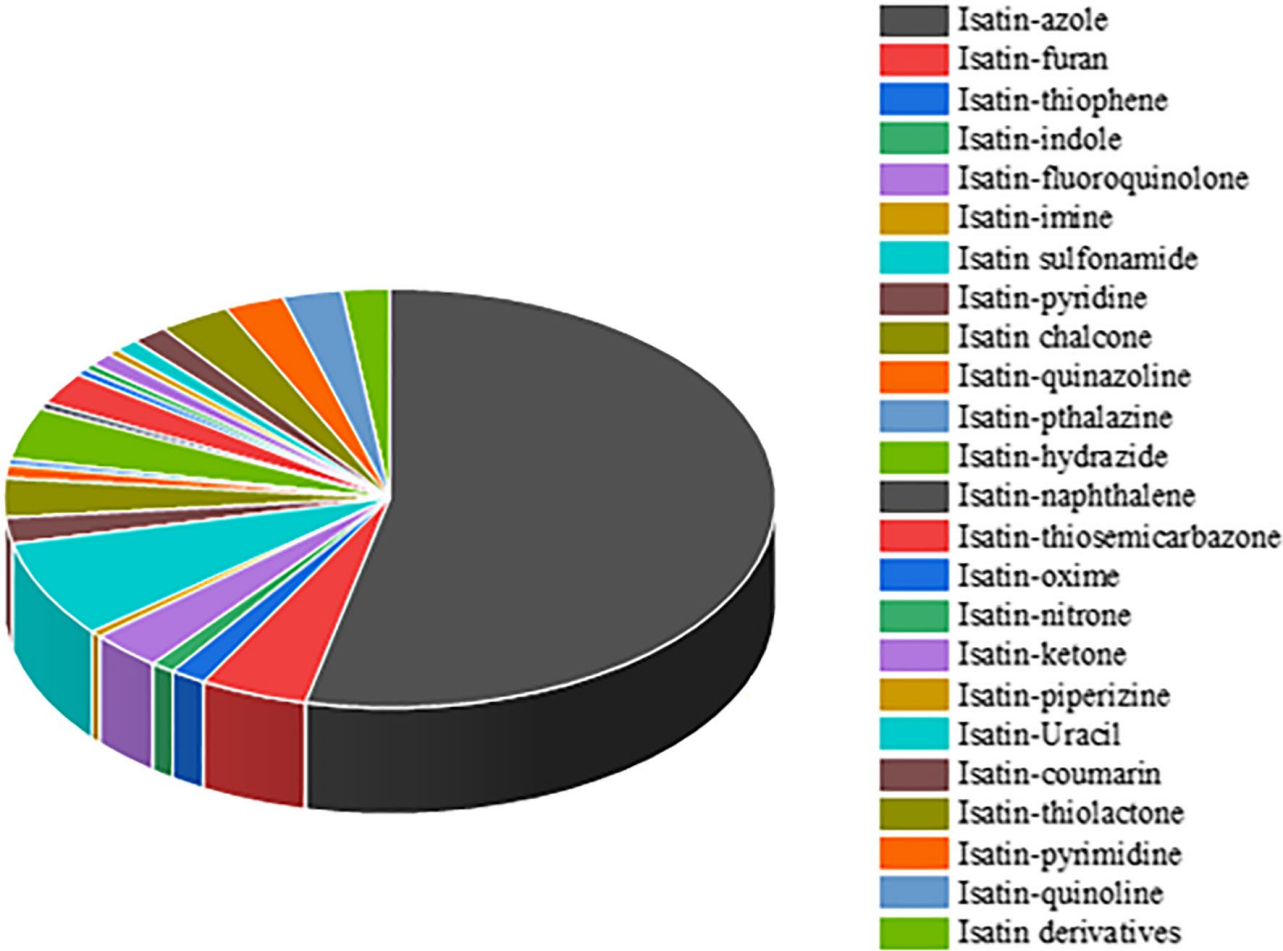
Distribution of isatin hybrids

Recently, some isatin-containing compounds have been approved for clinical trials (Sunitinib and Toceranib) [17] used in the treatment of tumors, while others (Nintedanib, Semaxinib, and Orantinib) are currently undergoing clinical trials for the evaluation of their therapeutic activities as anticancer agents [2]. This triple angiokinase inhibitor, Nintedanib, indicated for the treatment of idiopathic pulmonary fibrosis, systemic sclerosis-associated interstitial lung disease, and in combination with docetaxel for non-small cell lung cancer is a hybrid of indole and piperazine pharmacophores. Sunitinib, a receptor tyrosine kinase inhibitor, and chemotherapeutic agent is used for the treatment of renal cell carcinoma (RCC) and imatinib-resistant gastrointestinal stromal tumor (GIST) is a hybrid of isatin and pyrrole pharmacophores. The development of a single hybrid compound by combining two or more pharmacophores has been proven to be a promising approach in the development of new drugs that have the potential to overcome drug resistance and possess improved activity when compared to parent drugs [39]. It is therefore plausible that the molecular hybridization of the isatin moiety with other pharmacophores has the potential to generate new and more effective therapeutic candidates [8]. There exist several isatin hybrid molecules generated by the combination of isatin moiety with other useful pharmacophores that have outstanding biological activities. Some of these hybrids include Isatin-Azole hybrids [8–10, 14, 23, 40–45], Isatin-furan hybrids [9, 18, 40, 42, 46], Isatin-thiophene hybrids [8, 47], Isatin-indole hybrids [9, 48], Isatin-fluoroquinolone hybrids [9, 17, 49], Isatin-Imine hybrids[9], Isatin-sulfonamide hybrids [2, 9, 21, 50, 51], Isatin-pyridine hybrids [52–55], Isatin-chalcone hybrids [56], Isatin-quinazoline hybrids [57, 58], Isatin-phthalazine hybrids [57], Isatin-hydrazide hybrids [9, 40, 42, 55], Isatin-naphthalene hybrids [14], isatin-thiosemicarbazone hybrid[9, 20], Isatin-oxime hybrids [59], Isatin-nitrone hybrids [59], Isatin-ketone hybrids [60], Isatin-piperazine hybrids [61], Isatin-uracil hybrids [62], Isatin-coumarin hybrids [63], Isatin-thiolactone hybrids [64], and Isatin-pyrimidine hybrids. Figure 2 presents a pie chart illustrating how these isatin hybrids are distributed (further details on the structures of these hybrids can be found in the supplementary data) [17]. The purpose of this review is to set up the direction for the design and development of isatin hybrids with tailored biological properties as effective therapeutic candidates inspired by nature.

## Isatin-azole hybrids

**Fig. 3:**
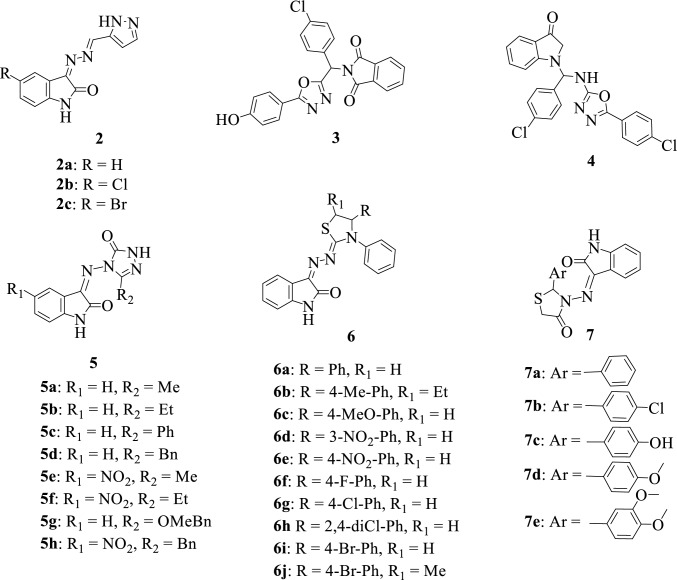
Chemical structures of isatin-azole hybrids

Azole, a privileged scaffold of choice when designing novel therapeutic agents, is mainly found as core structure in several natural products and synthesized compounds that are used by pharmaceutical or agrochemical industries [65]. Most azole compounds are used as antifungal drugs [66, 67] and some of its derivatives possess a variety of biological properties such as anticancer [7, 66], antibacterial [67, 68], and antitubercular properties [17, 69]. Several isatin-azole hybrids have been synthesized [4, 17, 28, 70–75] and reported to possess diverse pharmacological properties. The chemical structures of these isatin-azole hybrids are presented in Fig. 3. Eldehna et al. in 2018 [76] reported the synthesis of the isatin-pyrazole hybrids **2a-c** and evaluated their antiproliferative properties. The hybrid **2b** was identified as the most active analog portraying broad-spectrum activity against breast, colon, and lung human cancer cell lines with an average IC_50_ value of 2.14 μM. SAR studies revealed that the 5-pyrazolyl moiety was crucial and played an important role in the enhanced activity of this compound.

With the outbreak of SARS-CoV-2 and the urgent need for the development of bioactive molecules, Badavath et al. in 2020 [14] conducted in silico studies, by making use of computer-aided drug design approaches to screen over 118 compounds. The molecular docking studies against M^pro^ protein revealed that the isatin-oxidiazole hybrids **3** and **4** possessed excessive interactions with M^pro^ with best docking scores of − 11.22 and − 11.15 kcal/mol, respectively. These hybrids were composed of a central carbon atom bearing three different ring systems permitting them to make multiple interactions with the binding pocket of M^pro^. Thus, they could serve as starting points for the development of potential SARS-CoV-2 M^pro^ inhibitors. Özil et al. in 2011 [77] synthesized a series of isatin-1,2,4-triazole hybrids **5a-h** and evaluated their antimicrobial properties against four bacterial strains *Escherichia coli*, *Pseudomonas aeruginosa*, *Staphylococcus aureus*, and *Bacillus subtilis*. Hybrid **5 g** emerged with quite interesting antibacterial activity against *Staphylococcus aureus* and *Bacillus subtilis* bearing a minimal inhibitory concentration (MIC) value of 8 and 16 µg mL^−1^, respectively. Triazole derivatives which are known to possess antifungal activity in this case played an essential role in enhancing the antibacterial activity of this compound against the tested gram-positive bacteria. Neglected tropical diseases remain a global threat to health and thus there is a need for the development of new approaches and therapies to fight against these infections. Freitas et al. in 2021 [43] reported the synthesis and evaluation of the antiparasitic properties of some isatin-thiazolyl **6a-6j** hybrids. The hybrids **6e, 6 h, 6i,** and **6j** were found to be the most potent compounds with anti-*Trypanosoma cruzi* activity for trypomastigote form having IC_50_ values of 4.43 μM, 2.05 μM, 4.12 μM, and 1.72 μM, respectively. Nikalje et al., 2015 [10] described the microwave-assisted synthesis of a series of novel isatin-thiazolidin-4-one hybrids **7a-e** and analyzed their anticonvulsant activities in mice using maximal electroshock seizure (MES) and subcutaneous pentylenetetrazole (sc-PTZ)-induced seizure tests. Hybrids **7c** and **7e** with small electron-donating polar groups at the para-position of the phenyl ring exhibited potent protection against maximal electroshock seizure (MES) test cells thus indicating interesting anticonvulsant properties [28].

## Isatin-furan hybrids

**Fig. 4:**
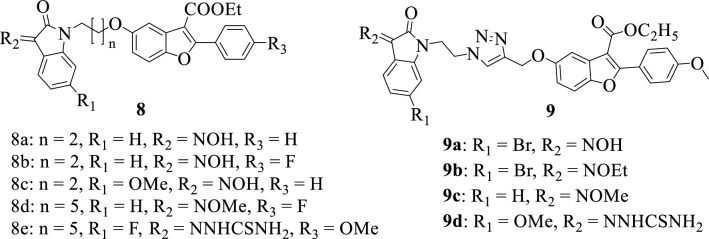
Chemical structures of isatin-furan hybrids

Furan is an important pharmacophore of natural origin with several biological properties (anticancer, antimalarial, antibacterial, and antifungal). It has been used as a starting material in the production of several industrial chemicals such as catalysts, resins, agrochemicals, and pharmaceuticals [78, 79]. The chemical structures of isatin-furan hybrids are presented in Fig. 4. The synthesis and antibacterial evaluation of a series of isatin-benzofuran hybrids **8a-e** were reported by Gao et al., in 2019 [18]. The synthesized compounds were tested on a panel of gram-negative and gram-positive bacteria and the MIC values were obtained. The hybrid **8e** was identified as the most promising compound with interesting antibacterial activity against majority of the tested pathogens *(Staphylococcus epidermidis, Staphylococcus aureus, Enterococcus faecalis, Enterococcus faecium, Escherichia coli, Klebsiella pneumoniae, Pseudomonas aeruginosa, Enterobacter aerogenes, Proteus mirabilis*) with MIC values of < 1 μg/mL. SAR demonstrated that incorporating a thiosemicarbazide at position C-3 of the isatin moiety as well as an electron-withdrawing group at position C-5 enhanced the activity of the compound. In 2018, Gao et al., [80] reported the synthesis of some isatin-benzofuran hybrids 9a-d and evaluated their antimycobacterial activity against *Mycobacterium tuberculosis* (MTB H37Rv strain) and MDR-TB (Multidrug-Resistant Tuberculosis) strains. Among the synthesized compounds, the hybrid **9d** was found to be the most active with over 128 folds effectiveness when compared to Rifampicin, a well-known antibiotic used in the treatment of tuberculosis having MIC values of 0.25 and 0.5 µg/mL against MTB H37Rv and MDR-TB strains, respectively. Results of SAR studies indicated that substituents at positions C-3 and C-5 of the isatin moiety play a vital role in the antimycobacterial activity of the compounds. The presence of an electron-donating group at C-5 and a hydrogen-bond donor group at C-3 accounts for the enhanced antimycobacterial activity of hybrid **9d** [42].

## Isatin-thiophene hybrids

**Fig. 5:**
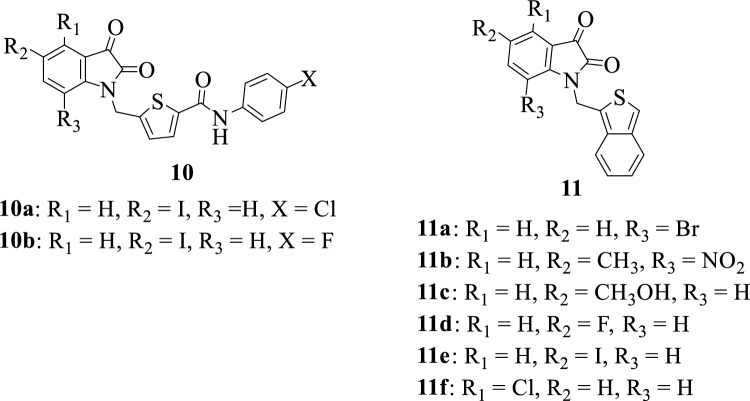
Chemical structures of isatin-thiophene hybrids

Thiophene, one of the most abundantly found heterocyclic rings present in biological systems, has emerged as a potent scaffold in drug discovery. This moiety and its derivatives have found widespread applications in different fields of life such as the pharmaceutical and dye industries. Several pharmacological properties have been reported to be associated with this scaffold, some of which include anticancer, antimicrobial, and anti-inflammatory properties [81, 82]. Figure 5 shows some of the chemical structures of isatin-thiophene hybrids. Chen et al. in 2005 [83] synthesized some isatin derivatives **10a-b** and **11a-f**. The synthesized compounds were evaluated in vitro for their inhibitory activity against SARS coronavirus 3CL protease. Notably, some of the synthesized compounds exhibited potent inhibitory activity against the virus with hybrids **11a** and **11e** being the most active hybrids among the compounds having IC_50_ values of 0.98 µM and 0.95 µM, respectively. The SAR studies suggested that the bioactivity of these compounds was greatly influenced by the nature of the substituents on the isatin moiety and the sidechain [14, 47].

## Isatin-indole hybrids

**Fig. 6:**
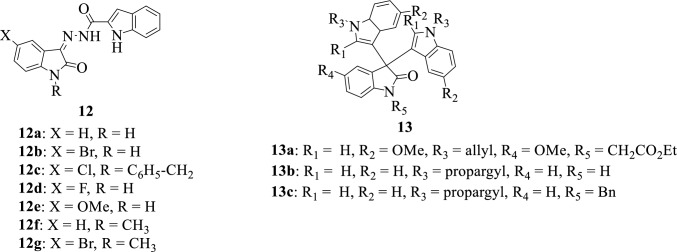
Chemical structures of isatin-indole hybrids

Indoles constitute an important subunit for the discovery of new drug candidates. It is widely distributed in natural products and bioactive molecules and is responsible for the fecal smell in human feces, scents of flowers, and the flowery smell of perfumes [84–86]. The indole moiety is a versatile molecule with several biological properties such as antifungal, antimicrobial, antiviral, and antitubercular properties [87]. Figure 6 presents some of the chemical structures of isatin-indole hybrids.

Al-wabli et al. in 2020 [48] reported the synthesis of some isatin-indole molecular hybrids **12a-g** and evaluation of their properties as antiproliferative agents against human breast (ZR-75), colon (HT-29), and lung (A-549) tumor cell line. The hybrid **12c** showed potent antiproliferative activity with an IC_50_ value of 1.17 µM which was approximately sevenfold greater than Sunitinib, a well-known anticancer medication. The SAR studies revealed that hybrids bearing *N*-benzyl moiety on isatin were more active with better antiproliferative activity. Some bis-isatin-indole hybrids **13a-c** were synthesized and reported by Praveen in 2011 [88]. The anticonvulsant and antibacterial properties of the synthesized compounds were evaluated against Maxima Electroshock seizure (MES) model and two bacterial strains: *Staphylococcus aureus* and *Escherichia coli*, respectively. The hybrids **13b** and **13c** demonstrated excellent anticonvulsant activity and in addition, hybrid **13c** revealed excellent antibacterial activity against *Escherichia coli*. Results from SAR demonstrated that the replacement of the *N*-allyl group (**13a**) with a propargyl group (**13b** and **c**) resulted in a remarkable improvement in the activity of these compounds [9].

## Isatin-fluoroquinolone hybrids

**Fig. 7:**
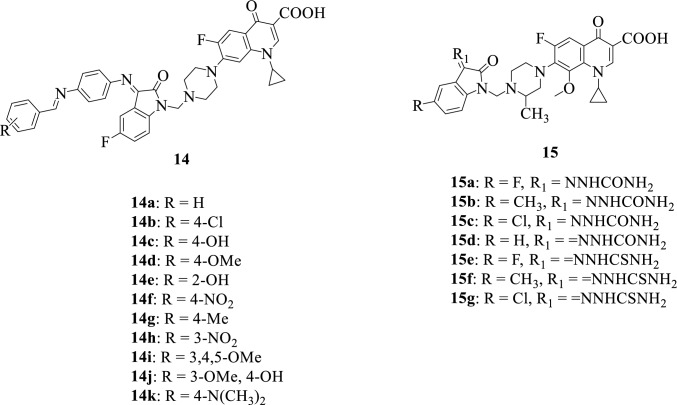
Chemical structures of isatin-fluoroquinolone hybrids

Quinolone is an essential class of nitrogen-containing heterocycles widely used as a building block for medicinal agents. Fluoroquinolones possess a broad-spectrum activity and very good oral bioavailability, and as such are often used as antibacterial agents. Some fluoroquinolones which are currently available include Ciprofloxacin, Gemifloxacin, Levofloxacin, Moxifloxacin, Norfloxacin, and Ofloxacin [19, 89–91]. Figure 7 presents some of the chemical structures of isatin-fluoroquinolone hybrids.

In 2013, to develop potential antimicrobials, Prakash et al. [92] reported the synthesis of a series of novel Ciprofloxacin-isatin hybrids **14a-k**. Most of the compounds showed interesting in vitro antibacterial and antifungal activity against the investigated microbes. The hybrid **14c** was identified as the most potent hybrid with better antibacterial activity against *Staphylococcus. aureus*, *Escherichia coli*, and *Pseudomonas aeruginosa* when compared to the parent drug Ciprofloxacin, and similar antifungal activity against *Aspergillus fumigatus* and *Aspergillus niger* when compared to Ketoconazole. The presence of the electron-donating substituent (-OH) plays a crucial role in improving the antibacterial activity of this compound [9].

Over one-third of the world’s population is potentially infected with tuberculosis (TB), a common infectious disease. In the quest for novel, effective, and fast-acting anti-TB drugs with low toxicity, Sriram et al. in 2006 [93] synthesized a series of Gatifloxacin-isatin **15a-g** hybrids and evaluated their antimycobacterial activity. Hybrid **15d** was shown to be the most potent with improved activity when compared to the parent drug Gatifloxacin with an IC_50_ value of 3.0 µg/mL. Fluoroquinolones play an essential role in the penetrative ability of compounds across cells leading to the assumption that penetration is pivotal for antimycobacterial activity of quinolones. Bearing this in mind, SAR studies illustrated that increasing the lipophilic character of the compounds at position C-7 resulted in an increase in activity [17].

## Isatin-sulfonamide hybrids

**Fig. 8:**
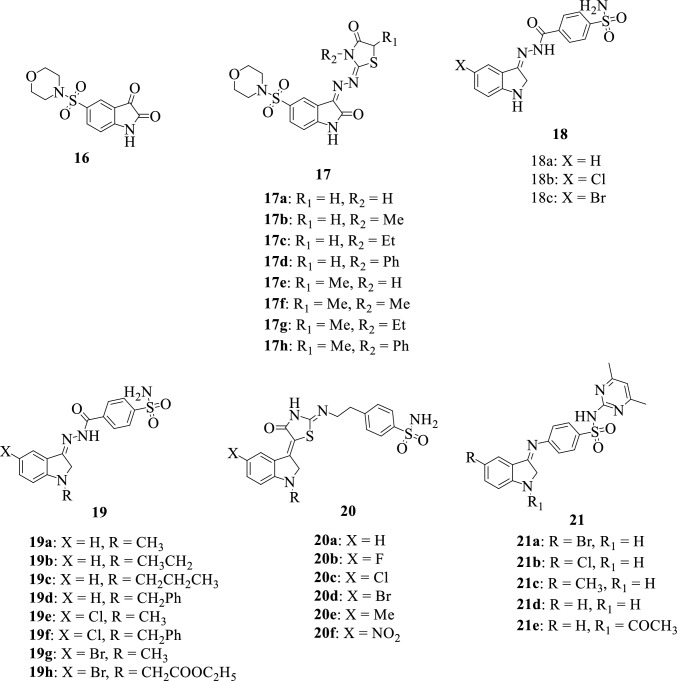
Chemical structures of isatin-sulfonamide hybrids

Sulfonamides are naturally occurring structural motifs in medicinal chemistry with leading roles in novel drug design and development against complex infections [91]. They are highly versatile organo-sulfur compounds containing the -SO_2_NH_2_ and/or -SO_2_NH- groups and small chemical modifications often result in improved activity. Sulfonamides are generally used in the treatment of bacterial infections and possess several biological activities such as antifungal, anti-inflammatory, antioxidant, diuretic, and anticancer [98, 99]. The chemical structures of these isatin-sulfonamide hybrids are presented in Fig. 8.

In 2014, Farag [94] reported the synthesis and evaluation of antimicrobial activity of a series of 5-(morpholinosulfonyl)isatin hybrids **16** and **17a-h**. The synthesized compounds were evaluated for their activity against gram + ve (*Staphylococcus aureus*, *Staphylococcus epidermidis*, and *Bacillus subtilis*), gram –ve (*Proteus vulgaris*, *Klebsiella pneumonia*, *Shigella flexneri*) bacteria, and fungi. Hybrid **16** revealed better antibacterial activity against all tested bacteria strains (MIC: 0.007–0.49 μg/Ml) when compared to Ampicillin B and fourfold antifungal potency against *Aspergillus fumigatus* when compared to Amphotricin B with an MIC value of 0.24 µg/mL. SAR revealed that the oxygen at position C-3 is crucial for activity and the replacement of oxygen with other substituents had detrimental impacts on the activity of the compounds.

Abo-Ashour et al., [95] with the main goal of developing novel isatin-based anticancer candidates targeting the tumor-associated hCA isoforms IX and XII, synthesized two series of isatin-sulfonamide hybrids **18** and **19a-h** followed by the evaluation of their in vitro biological activity. All the synthesized compounds revealed potent inhibitory activities against the tested hCA isoforms and thus were further investigated for their antiproliferative activity against several cancer cell lines. Notably, the hybrids **19f** and **19 h** were the most active against the various cell lines inhibiting the cancer cells in a concentration-dependent manner [50].

Eldehna et al., 2018 [96] synthesized and evaluated the anticancer activity of a series of isatin-sulfonamide hybrids **20a-f** against colorectal cancer (HCT-116) and breast cancer (MCF-7) cell lines. The most promising hybrid among the series **20e** exhibited potent anticancer activity against colorectal cancer (HCT-116) cell lines with an IC_50_ value of 3.67 ± 0.33 µM. Inhibitory activities of these compounds were influenced by the nature of the substituent inserted at position C-5 of the isatin moiety. An improvement in activity was observed with compounds bearing electron-donating groups at that position, while those with electron-withdrawing groups at C-5 possessed slightly reduced activity.

Selvam and collaborators in 2010 [51] reported the synthesis of a series of isatin-sulfadimidine hybrids **21a-e** and the determination of their antiviral activity against swine influenza A/California/07/2009 (H1N1) virus. The synthesized compounds revealed quite potent activity against the virus by blocking its adsorption to cells with the hybrids **21a** and **d** being the most active among the synthesized compounds.

## Isatin-pyridine hybrids

**Fig. 9:**
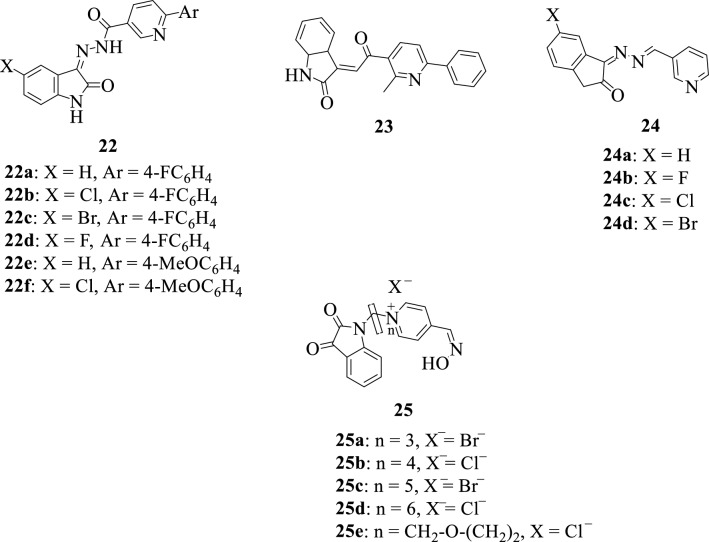
Chemical structures of isatin-pyridine hybrids

Pyridines are a class of heterocyclic nitrogenous compounds with tremendous applications in diverse fields of life. This moiety and its derivatives are naturally present in different molecules such as vitamins, co-enzymes, and alkaloids. Due to their wide range of pharmacological properties, pyridine-based compounds have found widespread applications in the field of drug design and discovery. It is widely used as a solvent for organic reactions, paints, and pharmaceuticals as well as intermediates in the manufacture of agrochemicals and pharmaceuticals [97]. The chemical structures of these isatin-pyridine hybrids are illustrated in Fig. 9.

Adopting a hybrid-pharmacophore approach, Eldehna et al. in 2014 [52] designed, synthesized, and evaluated the antiproliferative activity of a series of isatin-pyridine hybrids **22–24** against HepG2, A549 (lung), and MCF-7 (breast) cancer cell lines. Notably, hybrid **23** was identified as the most active compound with an over 2.7-fold increase in activity against HepG2 cell line when compared to Doxorubicin, a known anticancer medication. Quantitative structure–activity relationship studies revealed that the introduction of a more lipophilic and the bulky chlorine atom resulted in a tremendous increase in activity thus making hybrid **24c** the most active against A549 (lung) and MCF-7 (breast) cancer cell lines.

Kitagawa et al. in 2021 [53], in an attempt to combat organophosphorus poisoning caused by some pesticides and nerve agents, designed and synthesized a series of isatin-pyridine oxime hybrids **25a-e** and analyzed their properties as acetylcholinesterase reactivators. All the synthesized compounds demonstrated reactivation properties with hybrids **25c** and **25e** showing the highest percentage of reactivation even at low concentrations thus making them potential lead compounds. The SAR of hybrids **25a-e** suggested that the linker 1,5-pentanediyl (**25c**) is vital and plays an important role in the interaction of the compounds with the AChE binding sites.

## Isatin-chalcone hybrids

**Fig. 10:**
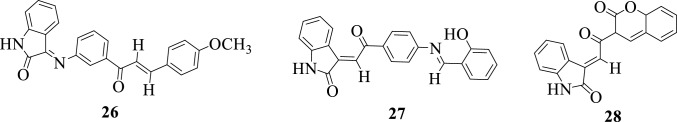
Chemical structures of isatin-chalcone hybrids

Chalcones are one of the most important classes of natural products derived from plants with widespread distribution in vegetables, teas, fruits, and many others [135, 136]. They are a group of plant-derived polyphenolic compounds, known to be biogenetic precursors of flavonoids and isoflavonoids with several medicinal and pharmaceutical applications some of which include antihypertensive, antibacterial, antiobesity, antimalarial, antiretroviral, anticancer, fungicidal, germicidal, herbicidal, and insecticidal [100, 101]. Figure 10 shows some of the chemical structures of isatin-chalcone hybrids.

Fayed et al., in 2021 [56] reported the synthesis and screening of a series of isatin-chalcone hybrids **26–28** for their anticancer activities against MCF-7 (breast), HepG-2 (liver), and HCT-116 (colon) human cell lines. All the synthesized compounds demonstrated quite interesting antitumor properties with the hybrid **27** showing very high anticancer activity against HepG-2 cell line with an IC_50_ value of 5.33 µM/mL when compared to Imatinib.

## Isatin-quinazoline hybrids

**Fig. 11:**
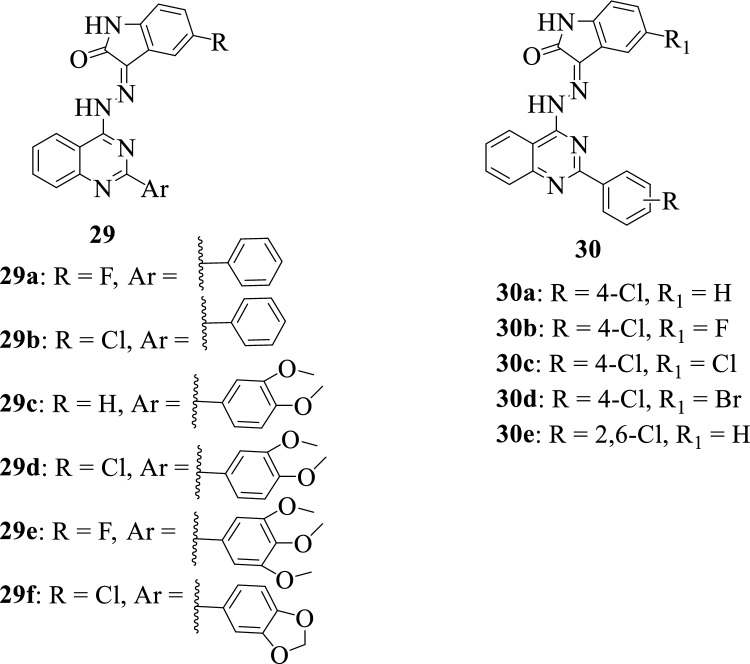
Chemical structures of isatin-quinazoline hybrids

Quinazoline scaffold is a vital class of biologically active nitrogen-containing heterocycles with unique properties such as ease of synthetic accessibility and flexible structural modification, which have motivated the exploitation of their biological activities [102]. This scaffold has attracted significant attention over the past years due to its diverse pharmacological activities such as antimalarial, anticancer, anticonvulsant, and anti-inflammatory properties [103]. The chemical structures of some selected isatin-quinazoline hybrids are displayed in Fig. 11.

Implementing a molecular hybridization approach, Fares et al., 2015 [58] designed and synthesized a series of isatin-quinazoline hybrids **29a-f**. The synthesized compounds were tested for their in vitro anticancer activity against liver, breast, and colon cancer cell lines. It is worth noting that, the hybrids **29a**, **29c,** and **29f** were the most active with the ability to induce apoptosis in liver HepG2 cells having IC_50_ values of 1.0 ± 0.2, 1.8 ± 0.4, and 2.4 ± 0.4 µM, respectively.

Eldehna et al. in 2017 [57] with the primary goal of developing potent antiproliferative agents capable of targeting triple-negative breast cancer (TNBC) MDA-MB-231 cell lines synthesized a series of isatin-quinazoline hybrids **30a-e**. The hybrid **30e** was found to be the most potent against MDA-MB-231 cell lines with over a 2.37-fold increase in activity when compared to 5-Fluorouracil, the reference drug. The anticancer SAR of hybrids **30** indicated that the introduction of the 2,6-dichloro substituent (**30e**) was beneficial for activity and could be assigned to its ability to increase the lipophilic character of the compound.

## Isatin-phthalazine hybrids

**Fig. 12:**
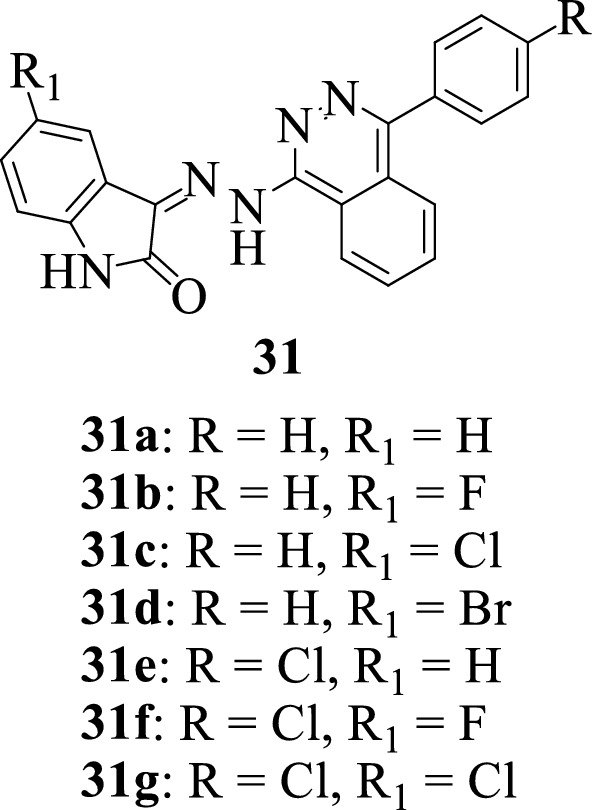
Chemical structures of isatin-phthalazine hybrids

Phthalazines are essential nitrogen-containing heterocyclic compounds with interesting chemical, industrial, and pharmacological properties such as anticancer, anticonvulsant, anti-inflammatory, antifungal, and antibacterial properties. Different drug molecules are presently available in the market which contain the phthalazine pharmacophore some of which include Hydralazine, Budralazine, Vatalanib, Olaparib, and Azelastine. Owing to its broad application in the treatment of diverse infections, the phthalazine scaffold has received much attention in the area of drug discovery. Phthalazines are used as starting materials for the development of new medications and as an intermediary in the synthesis of chemicals [104]. Figure 12 shows some of the chemical structures of isatin-phthalazine hybrids.

Exploring the potentials in the hybrid-pharmacophore approach, Eldehna et al., 2017 [57] reported the synthesis of a series of isatin-phthalazine hybrids **31a-g** and evaluated their activity as antiproliferative agents against triple-negative breast cancer (TNBC) MDA-MB-231 cell lines. Notably, the hybrid **31 g** showed improved activity against MDA-MB-231 (breast) cell lines with over a 2.44-fold increase in activity when compared to 5-Fluorouracil, the reference drug. SAR studies revealed that inserting substituents at the 4-phenyl group of the hybrids resulted in compounds with improved activity when compared to the unsubstituted compounds.

## Isatin-hydrazide hybrids

**Fig. 13:**
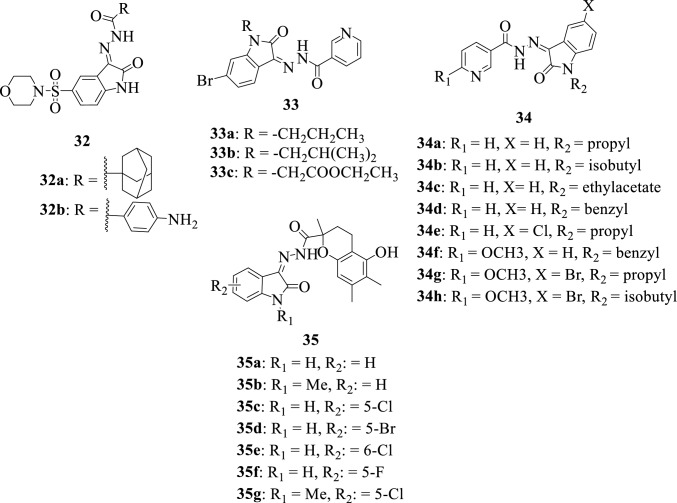
Chemical structures of isatin-hydrazide hybrids

Hydrazides represent an important class of organic compounds that contain the azomethine functional group connected to a carbonyl group. These functionalities accord the pharmacophore its unique pharmacological properties thus making it a key intermediate and vital starting material for the development of novel bioactive compounds. Several drugs are currently in use that contain the hydrazide moiety some of which include Isoniazid (antituberculosis), Nifuroxazide (antibiotic), Isocarbazide (antidepressant), Iproniazid (antituberculosis), and Galavit (anti-inflammatory) [105, 106]. The chemical structures of these isatin-hydrazide hybrids are presented in Fig. 13.

In 2020, Salem et al. reported the synthesis of some isatin-carbohydrazide hybrids **32a-b** and further evaluated their in vitro antimicrobial activity [107]. The compounds were tested on some strains of both gram-positive and gram-negative bacteria and the hybrid **32b** was found to possess the most potent antibacterial activity among the synthesized compounds with its activity comparable to that of Norfloxacin and Tetracycline. Antibacterial SAR evaluation of the hybrids demonstrated that the presence of the *p*-amino benzoic acid moiety in **32b** greatly influenced the increase in bioactivity of this hybrid.

Elsayed et al., 2021 [55] reported the synthesis of a series of isatin-nicotinohydrazide hybrids **33a-c** and **34a-h** followed by the evaluation of their activities as antitubercular and antibacterial agents. Among the synthesized compounds, the hybrids **34 g** and **34 h** were found to be the most potent antitubercular agents demonstrating broad-spectrum antibacterial activity against the tested strains, and SAR indicated that *N*-benzylation/methylation of the isatin moiety plays a pivotal role in exertion of the biological properties [42].

Rawat et al., 2016 [108] reported the synthesis of a series of isatin-carbohydrazide hybrids **35a-g** and the evaluation of their antimicrobial activity against different bacterial and fungal strains. Most of the synthesized compounds revealed interesting antimicrobial activities with the hybrids **35c** and **35d** being the most potent against the bacterial strain *Escherichia coli*, while hybrids **35a** and **35b** revealed very potent antifungal activity against *Candida albicans*.

## Isatin-thiosemicarbazone hybrids

**Fig. 14:**
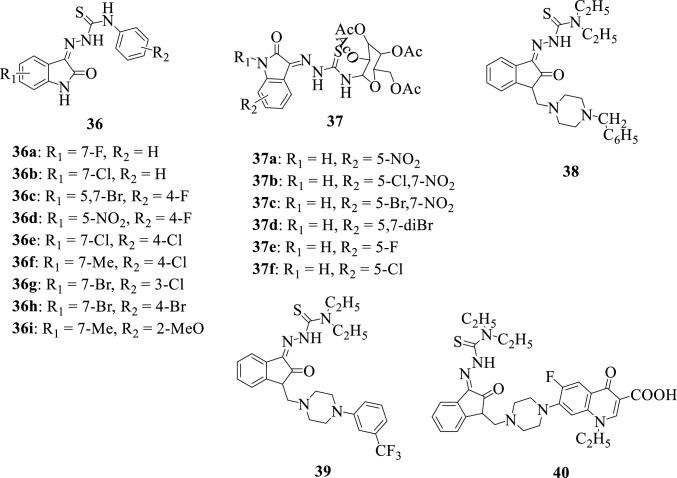
Chemical structures of isatin-thiosemicarbazone hybrids

Thiosemicarbazones are an important class of ligands generally obtained as condensation products from the reaction of thiosemicarbazide with aldehydes and ketones. Over the years, thiosemicarbazones have gained so much interest owing to their metal-chelating properties, wide range of biological properties, and structural flexibility [109]. Figure 14 shows some of the chemical structures of isatin-thiosemicarbazone hybrids.

To discover novel anti-methicillin-resistant *Staphylococcus aureus* (MRSA) agents, Zhang et al., 2015 [137] synthesized a series of isatin-β-thiosemicarbazones hybrids **36a-36i**. The synthesized compounds were evaluated for their antibacterial activity against gram-positive bacterial strains: *Staphylococcus aureus* (ATCC 6538) and *Bacillus subtilis* (ATCC 6633). All tested compounds exhibited interesting antibacterial activity with **36b** being the most active with a minimum inhibitory concentration (MIC) value of ≤ 1.56 mg/L against the tested strains, and SAR studies revealed that the insertion of a halogen at position-7 of isatin is an essential structural modification required to obtain favorable antibacterial activity.

Thanh et al., 2016 [20] reported the synthesis and evaluation of the in vivo antioxidant/in vitro antimicrobial activity of a series of isatin-thiosemicarbazone hybrids **37a-f**. The in vitro antimicrobial activity was conducted against different bacterial (*Staphylococcus aureus*, *Escherichia coli*, *Klebsiella pneumonia*, *Pseudomonas aeruginosa*, *Staphylococcus epidermidis*, *Bacillus subtilis*, *Enterobacter aerogenes*) and fungal strains (*Aspergillus niger*, *Candida albicans*, *Fusarium oxysporum*, *Saccharomyces cerevisiae*), while in vivo antioxidant activity was determined by evaluating the superoxide dismutase (SOD), glutathione peroxidase (GSH-Px), and catalase (CAT) activities of the compounds. The synthesized compounds revealed quite promising activities and the hybrid **37d** was identified as the most potent antioxidant, antibacterial, and antifungal agent.

Conducting pharmacophoric modeling studies on non-nucleoside reverse transcriptase inhibitors (NNRTIs), a series of isatin-β-thiosemicarbazone hybrids **38–40** were synthesized and evaluated for their anti-HIV activity. The synthesized hybrids were found to possess interesting anti-HIV activity with hybrid **39** being the most active among the synthesized compounds with an EC_50_ value of 2.62 µM [15].

## Isatin-oxime hybrids

**Fig. 15:**
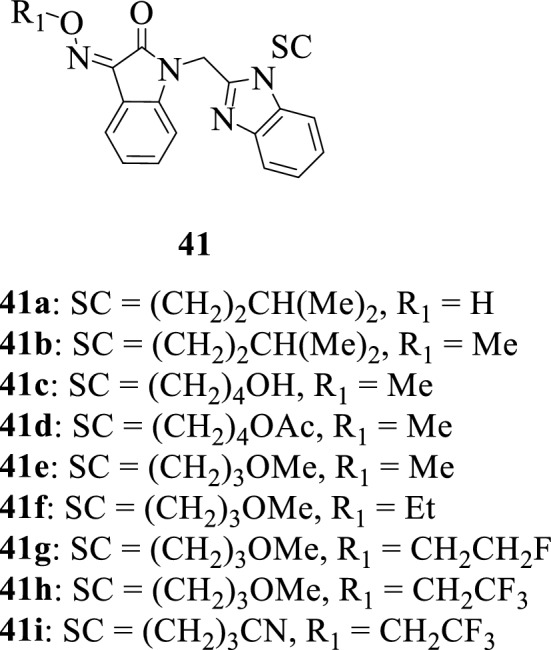
Chemical structures of isatin-oxime hybrids

Oximes are an essential class of nitrogen-containing compounds usually obtained as condensation products from the reaction of hydroxyl amines with aldehydes or ketones. This pharmacophore has found widespread use in different fields of life such as in industries, some oxime-containing compounds are used as artificial sweeteners. A good number of marketed drugs contain the oxime moiety some of which include Pyraloxime methiodine: a cholinesterase inhibitor and Ceftobiprole [110, 111]. Furthermore, oxime-containing chemicals have been reported to possess antiviral properties against influenza virus A and HIV-1 virus as well as anticancer properties against human breast and colon adenocarcinoma cell lines [112, 113]. The chemical structures of these isatin-oxime hybrids are presented in Fig. 15.

To meet the demand for orally active inhibitors of respiratory syncytial virus (RSV) replication, Sin et al., 2009 [59] synthesized a series of isatin hybrids **41a–41i**. The tested compounds revealed potent antiviral activities with the hybrids **41b–41g** bearing methyl, ethyl, and fluoroethyl substituents being the most active hybrids, and SAR studies revealed these small oxime substituents are preferable for antiviral activity.

## Isatin-nitrone hybrids

**Fig. 16:**
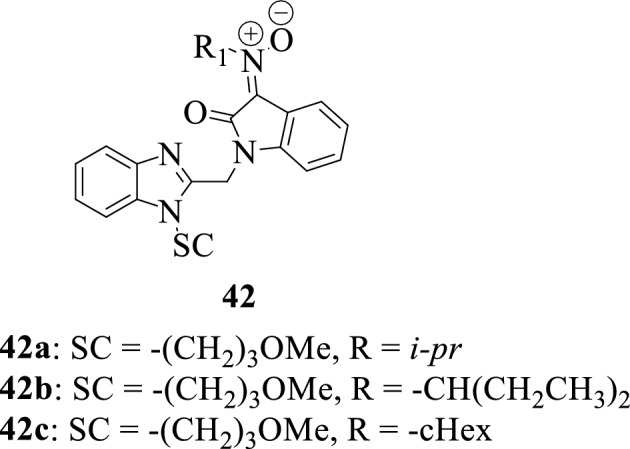
Chemical structures of isatin-nitrone hybrids

Nitrones are organic species that react with, “trap” and stabilize free radicals for identification and characterization purposes [114]. They are potent antioxidant molecules capable of reducing oxidative stress as well as suppressing signal transduction processes suggesting potential anti-inflammatory and antiapoptotic activities [115–117]. The chemical structures of these isatin-nitrone hybrids are shown in Fig. 16.

Sin et al., 2009 [59] reported the synthesis of a series of isatin-nitrone hybrids **42a–42c** and the evaluation of their inhibitory activity against respiratory syncytial virus (RSV). The synthesized compounds revealed moderate antiviral activity with the hybrid **42c** being the most potent.

## Isatin-piperazine hybrids

**Fig. 17:**
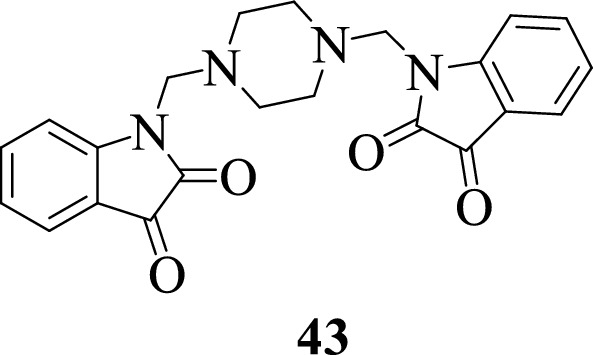
Chemical structure of an isatin-piperazine hybrid

Piperazine is a vital heterocyclic scaffold found in several biologically active compounds. This scaffold is present in some antiviral agents such as Delavirdine and Indinavir, which are used in HIV treatment. It is considered a privileged scaffold for drug design and widely used due to its unique properties some of which include solubility, basicity, chemical reactivity, and conformational properties [118, 119]. This ring is present in several commercially available drugs and its derivatives are known to possess a broad spectrum of therapeutic properties such as antidepressant, anticancer, antimalarial, anticonvulsant, antifungal, and antitubercular properties [120]. The chemical structure of an isatin-piperazine hybrid is shown in Fig. 17.

In 2021, Omar et al. [61] in the quest for possible SARS-CoV-2 Protease Enzyme inhibitors synthesized the isatin-piperazine hybrid **43** and evaluated its physicochemical, bioactivity scores, and pharmacokinetic properties using in silico computational tools. Molecular docking studies were conducted to predict the inhibitory activity of the ligand against the SARS-CoV-2 main protease enzyme. Based on the study, the piperazine ligand made strong hydrogen bonding interactions with the SARS-CoV-2 Protease with a negative dock energy thus suggesting it could be a good lead for the design of new drug candidates.

## Isatin-uracil hybrids

**Fig. 18:**
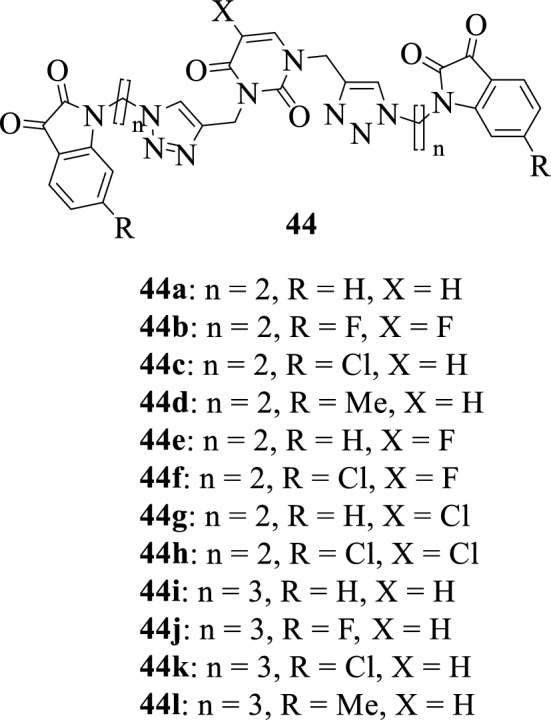
Chemical structures of isatin-uracil hybrids

Uracil, a naturally occurring pyrimidine nucleobase, is a major component of nucleic acid. Oxidative degradation of uracil yields urea and maleic acid in the presence of hydrogen peroxide and ferrous ions. It has widespread applications in different fields of life such as medicine, pesticide, and chemical synthesis. Uracil is commonly used as a starting material in the synthesis of many pyrimidine-based herbicides and the design and application of medicine [62]. The chemical structures of some isatin-uracil hybrids are shown in Fig. 18.

Kumar et al., 2012 [62] reported the synthesis of a series of isatin-uracil hybrids **44a–44 l** and evaluation of their cytotoxic activity against three human cancer cell lines HeLa (cervix), MCF-7 (breast), and DU145 (prostate). Among the synthesized compounds, the hybrids **44g** and **44k** were found to be active against DU145 (prostate) cancer cell lines at low concentrations. Notably, most of the compounds were inactive against the HeLa (cervix) cell line except for hybrids **44d** and **44h** bearing electron-withdrawing substituents. SAR studies identified two key factors that influence the activity of these hybrids, the presence of a halogen atom on Uracil and increasing the chain length from *n* = 2 to *n* = 3.

## Isatin-coumarin hybrids

**Fig. 19:**
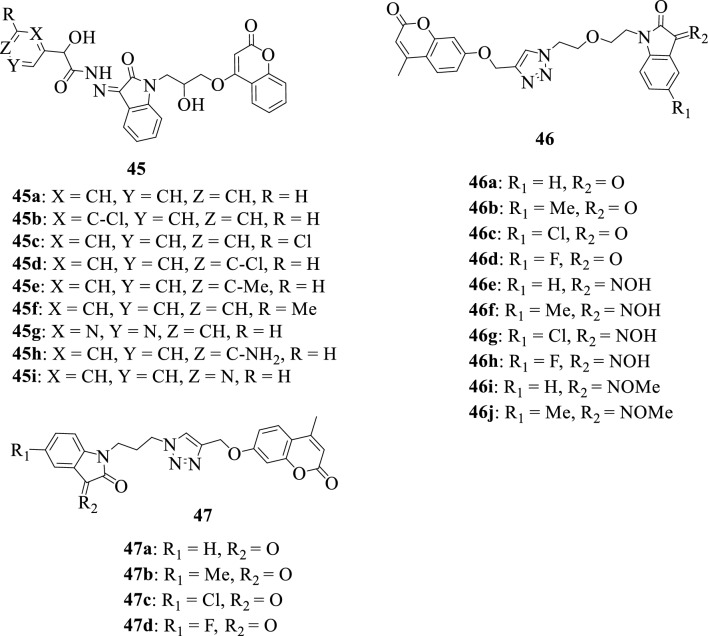
Chemical structures of isatin-coumarin hybrids

Coumarin represents a privileged scaffold for medicinal chemists with unique physicochemical properties that undergo easy synthetic transformations [121, 122]. It is found extensively in nature and its derivatives have been found to demonstrate interesting pharmacological activities (antibacterial, antifungal, antimalarial, and anticancer activities). Coumarins are widely used in perfumes, hand soap, detergents, and lotions where they function as fragrance enhancers or stabilizers [123, 124]. Figure 19 presents some of the chemical structures of isatin-coumarin hybrids.

Considering the availability of limited and unsatisfactory antileishmanial chemotherapeutics, Khatoon et al., in 2021 [63] synthesized a series of isatin-coumarin hybrids **45a–45i**. The synthesized compounds were evaluated for their in silico and in vitro activities against Leishmaniasis. Notably, hybrids **45f**, **45h,** and **45i** were found to be the most active at macro molar concentrations against *Leishmania tropica* promastigotes and amastigotes.

In 2019, Diao et al. [125] reported the design and synthesis of a series of isatin-coumarin hybrids **46a–46l**, and evaluation of their in vitro anticancer activities against HepG2 (liver carcinoma), Hela (cervical cancer), A549 (lung adenocarcinoma), DU145 (prostatic cancer), SKOV3 (ovarian carcinoma), MCF-7 (breast cancer), and drug-resistant MCF-7/DOX (doxorubicin-resistant MCF-7) human cancer cell lines. The compounds revealed weak to moderate anticancer activities, and as such can be considered as starting points for further research. The anticancer SAR studies demonstrated that the nature of the substituents at positions C-3 and C-5 are vital for activity, as electron-donating substituents at C-5 enhanced activity while hydrogen-bond donor groups at C-3 are important for activity.

Huang et al., 2019 [126] reported the design, synthesis, and evaluation of the in vitro antitubercular activity of a series of isatin-coumarin hybrids **47a–47d** against MTB H37Rv. The compounds, however, were inactive but could serve as good starting points for the development of anti-TB molecules.

## Isatin-thiolactone hybrids

**Fig. 20:**
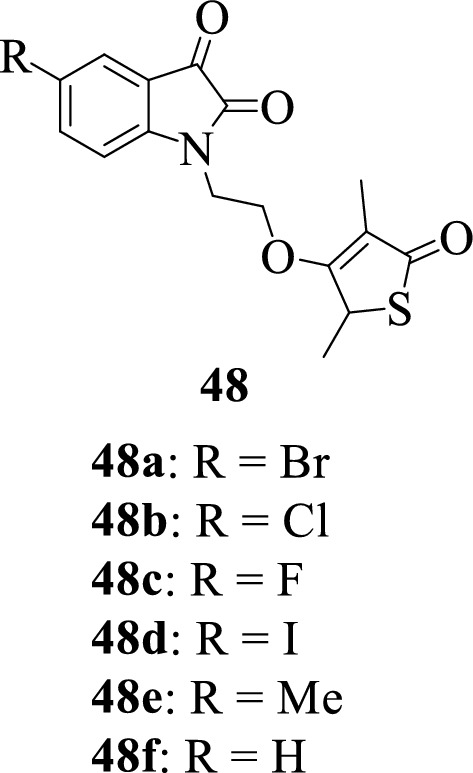
Chemical structures of isatin-thiolactone hybrids

Thiolactone is an essential class of heterocyclic scaffold with the extensive use of their cores as synthetic intermediates for the generation of ligands required for applications in catalysis and medicinal chemistry. They are often referred to as latent thiols and have been reported to possess anticancer, antibacterial, and anti-Alzheimer activity [127]. Figure 20 presents some of the chemical structures of isatin-thiolactone hybrids.

Hans et al., 2011 [64] synthesized and evaluated the antiplasmodial activity of a series of isatin-thiolactone hybrids **48a–f** against chloroquine-resistant (W2) strain of *Plasmodium falciparum*. Notably, none of the compounds revealed potent antimalarial activity. However, it was observed that the activity of some of the compounds was enhanced because of hybridization and could be a starting point for further investigation.

## Isatin-pyrimidine hybrids

**Fig. 21:**
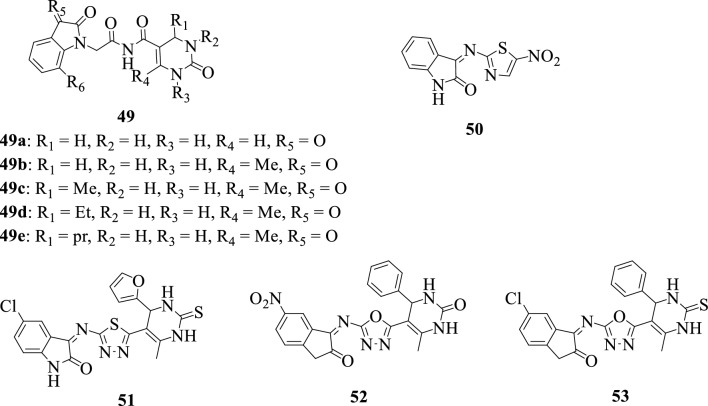
Chemical structures of isatin-pyrimidine hybrids

Pyrimidines represent one of the most active classes of compounds with a wide spectrum of biological activities that can be exploited for drug discovery [128]. Substituted pyrimidines are widely distributed in nature and are one of the first compounds that were studied by organic chemists. They can be found in both natural products (Vitamin B1) and synthetic compounds (Barbituric acid and Veranal) used as hypnotics [129]. The chemical structures of some isatin-pyrimidine hybrids are presented in Fig. 21.

In 2016, Devale et al. [54] reported the synthesis of a series of isatin-pyrimidine hybrids **49a–e**. These compounds were screened for their in vitro Reverse Transcriptase (RT) inhibitory activity against the HIV-1 virus, resulting in the identification of two hybrids **49c** and **49d** with higher RT inhibitory activity when compared to Rilpivirine, a reference drug. SAR studies revealed that the presence of aliphatic substituents rather than aromatic substituents at position R_1_ greatly favored the inhibitory activity of the compounds.

Akhaja et al., 2012 [130] reported the synthesis and in vitro evaluation of some isatin-pyrimidine hybrids **50–53** as antitubercular agents. Most of the synthesized compounds revealed moderate activity with the hybrids **50** and **51** being the most active against MTB H37Rv. Notably, hybrids **52** and **53** were found to completely inhibit MTB H37Rv by 99% at an MIC of 3.10–3.12 mg/mL.

## Isatin-quinoline hybrids

**Fig. 22:**
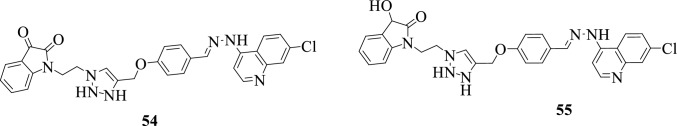
Chemical structures of isatin-quinoline hybrids

The quinoline moiety, a nitrogen-containing heterocyclic compound, can be found in several natural compounds. It is one of the most recognized fragments in bioactive compounds and is found in different pharmaceutically important alkaloids such as quinine and cinchonine. Pharmacological studies of quinoline have reported a broad spectrum of activities associated with this moiety [121, 122]. Figure 22 shows some of the chemical structures of isatin-quinoline hybrids.

Raj et al., 2014 [131] reported the synthesis and evaluation of antimalarial activity of two isatin-chloroquinoline hybrids **54** and **55** against chloroquine-resistant W2 strain of *Plasmodium falciparum*. The synthesized compounds were not as potent as standard antimalarial drugs. However, the most potent compound revealed activity that is comparable to that of Chloroquine thus suggesting these compounds could be a starting point for further research.

## Isatin-thioacetazone hybrids

**Fig. 23:**
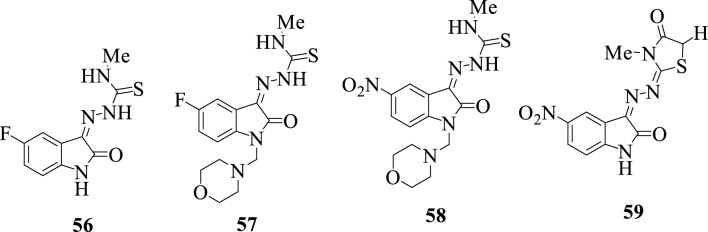
Chemical structures of isatin-thioacetazone hybrids

Thioacetazone is a bacteriostatic drug used in combination with other antimycobacterial agents to treat tuberculosis. However, the dermatological side effects associated with its use by AIDS patients have limited its exploitation. Thioacetazone has weak activity against MTB and is never used on its own. It is useful in preventing resistance to more powerful drugs like Isoniazid and Rifampicin [138]. The chemical structures of some isatin-thioacetazone hybrids are shown in Fig. 23.

To develop new and more potent antitubercular agents, a series of thioacetazone-isatin hybrids **56–59** were synthesized. Hybrid **57** revealed quite interesting inhibitory activity against MTB H37Rv, while hybrid **58** was found to be the least potent, and SAR revealed that halogenation at position C-5, as well as the insertion of a substituent at the N-1, influenced the antitubercular activity of the compounds [17].

## Other isatin hybrids

**Fig. 24:**
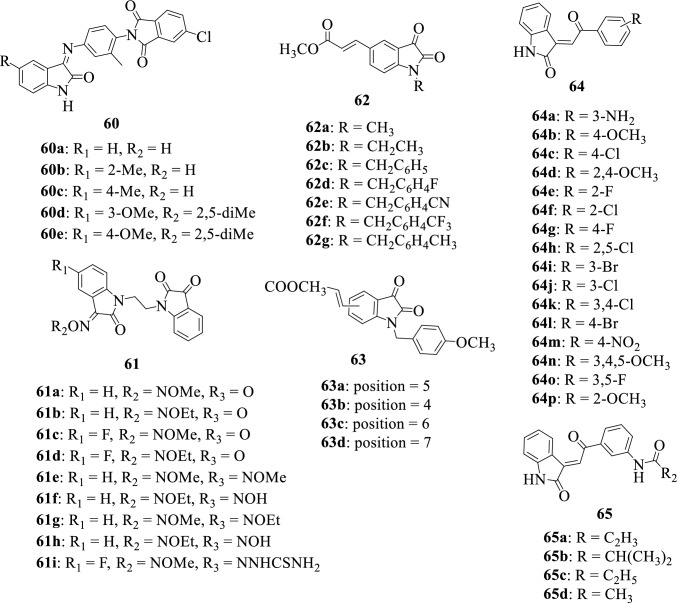
Chemical structures of other isatin hybrids

A series of isatin-imine **60a-60e** analogs were successfully synthesized and evaluated for their antibacterial and antifungal activities against certain microbes by Debnath et al., 2015. Some of the compounds portrayed quite interesting properties with **60d** being the most potent against the investigated microbes having the highest docking score. Structure–activity relationship studies revealed that the introduction of 2,5-dimethyl substituent at position R_2_ improved the activity of the compound [132]. Figure 24 presents some of the chemical structures of other isatin hybrids.

In 2018, Xu et al. [133] reported the synthesis of a series of ethylene tethered bis isatin derivatives **61a-i**. The synthesized compounds were evaluated for their in vitro antimycobacterial activities against MTB H37Rv and MDR-TB. All tested compounds revealed interesting antimycobacterial properties with **61i** being the most potent, and SAR illustrated that NNHCSNH_2_ at position C-3 and insertion of a halogen at C-5 greatly boosted the activity of this compound.

Teng et al., 2015 [134] reported the design and synthesis of a series of di- and tri-substituted isatin derivatives **62a-g** and **63a-d**, as well as the evaluation of their in vitro anticancer properties against human T-lymphocyte Jurkat cells. The compound **63a** was found to be the most potent compound capable of inhibiting the proliferation of Jurkat cells by inducing apoptosis with an IC_50_ value of 0.03 µΜ. SAR studies demonstrated that the combination of a 1-benzyl and 5-[trans-2-(methoxycarbonyl)ethen-1-yl] substitution results in improved cytotoxic activity.

Wang et al., 2018 [60] while attempting to exploit the potentials in molecular hybridization for the development of anticancer drugs synthesized some novel isatin-α,β-unsaturated ketone hybrids **64a-64 k** and **65a-65d**. Most of the synthesized compounds revealed potent antiproliferative properties in the tested cell line, and SAR revealed that the inhibition activity of the compounds greatly depended on the electron-withdrawing substituent on the benzyl ring. The hybrid **65a** was identified as the most potent hybrid which can be a promising lead compound for the development of anticancer agents.

## Conclusions

The isatin privileged scaffold can be found in a broad range of natural and synthetically derived pharmacologically active compounds having antibacterial, antifungal, antiviral, anticancer, anti-inflammatory, anticonvulsant, antitubercular, antiparasitic, and antioxidant properties. This review compiles published data on the synthesis and biological properties of some isatin hybrids as potential drug targets in an active area of medicinal chemistry. The literature survey demonstrated that the N‐1, C‐3, C‐4, C‐5, and C‐7 positions of the isatin scaffold can be modified, and the N‐1, C‐3, and C‐5 positions are much more favorable for modifications. In addition, the introduction of electron-withdrawing groups at positions 5, 6, and 7 of the indole rings can greatly increase the activities of the hybrids in comparison with isatin. However, the mono-substitution at the C-5 position can be considered most favorable since it is beneficial to control the electronic effect, lipophilicity, and physicochemical properties. For the N‐1 position, N-alkyl, -aryl, and -acyl substitutions are possible including azole. For C‐3 position, imine, hydrazone, and spiro‐ring are most common, but other pharmacophores, such as azole, are also tolerated. Among the isatin hybrids in this review, hybrids **2b**, **12c**, and **20e** showed interesting anticancer properties with IC_50_ values 2.14, 1.17 µM, and 3.67 ± 0.33 µM, respectively. Hybrids **5 g** and **8e** possessed promising antibacterial properties with MIC (minimal inhibition concentration) values 8 and < 1 µg/mL, respectively. Hybrids **7** and **13b** with the isatin moiety substituted at C-3 position expressed interesting anticonvulsant properties. Summarily, hybrids **6j** and **45f** showed antiparasitic properties, hybrids **9d** and **15d** antimycobacterial properties, hybrids **11e** and **21e** antiviral property, and hybrids **14c** and **16** antifungal properties. The compounds discussed in this review could serve as a starting point for further research on promising therapeutic drug candidates. Therefore, the concept of molecular hybridization with the possible modifications on the isatin moiety at the N-1, C-3, and C-5 positions can result in an array of compounds with diverse biological properties.

## Supplementary Information

Below is the link to the electronic supplementary material.

Supplementary file1 (DOCX 4071 KB)

## Data Availability

No datasets were generated or analyzed during the current study.

## Abbreviations

SAR: Structure–activity relationship
SARS-CoV-2: Severe acute respiratory syndrome coronavirus 2
IC_50_: Half maximal inhibitory concentration
MES: Maxima electroshock seizure
sc-PTZ: Subcutaneous pentylenetetrazole
hCA: Human carbonic anhydrase
AChE: Acetylcholinesterase
SOD: Superoxide dismutase
GSH-Px: Glutathione peroxidase
CAT: Catalase
NNRTIs: Non-nucleoside reverse transcriptase inhibitors
RSV: Respiratory syncytial virus
MTB H37Rv: *Mycobacterium tuberculosis* Strain H37Rv
MDR-TB: Multidrug-resistant tuberculosis
MIC: Minimum inhibitory concentration
RT: Reverse transcriptase
