# Supplementary material for: A survey of isatin hybrids and their biological properties
Source: Mol Divers. 2024 Jun 4;29(2):1737–60. doi: 10.1007/s11030-024-10883-z (PMC11909063; doi:10.1007/s11030-024-10883-z)
Supplement: Supplementary file 1 — Supplementary file1 (DOCX 4071 KB) [file 11030_2024_10883_MOESM1_ESM.docx]

**SUPPLEMENTARY DATA**

**A Survey of Isatin Hybrids and their Biological Properties**

**Vanessa Asoh Shu ^1^, Donatus Bekindaka Eni ^1,2^* and Fidele Ntie-kang ^1,2,3^***

^1^ Center for Drug Discovery, Faculty of Science, University of Buea, Buea, Cameroon. Email: [vanessaasoh1998@gmail.com](mailto:vanessaasoh1998@gmail.com) (SVA) ; [donatus.bekindaka@ubuea.cm](mailto:donatus.bekindaka@ubuea.cm) (DBE); [fidele.ntie-kang@ubuea.cm](mailto:fidele.ntie-kang@ubuea.cm) (FNK).

^2^ Department of Chemistry, Faculty of Science, University of Buea, Buea, Cameroon.

^3^ Institute of Pharmacy, Martin-Luther University Halle-Wittenberg, Halle (Saale), Germany.

|  |
| --- |

***** Correspondence: [donatus.bekindaka@ubuea.cm](mailto:donatus.bekindaka@ubuea.cm) (DBE); [fidele.ntie-kang@ubuea.cm](mailto:fidele.ntie-kang@ubuea.cm) (FNK).

**Table S1: Structure and biological activities of Isatin Hybrids**

| **S/N** | **Name of hybrid** | **Structure** | **IC_50_ Value** | **Activity** | **Reference** | |
| --- | --- | --- | --- | --- | --- | --- |
| 1 | Isatin | **** |  |  | [8] | |
| 2 | Isatin–pyrazole hybrids |  | 1.37–22.94 μM | Anticancer(lung, breast,colon cancer cells) | [8] | |
| 2a |  |  |  |  |  | |
| 2b |  |  |  |  |  | |
| **3** | Isatin-oxidiazole hybrids |  | Ki: 5.94 nM | Antiviral(SARS-Co-V-2 Mpro) | [14] | |
| **4** | Isatin-oxidiazole hybrids |  | Ki: 6.66 nm | Antiviral(SARS-Co-V-2 Mpro) | [14] | |
| **5** | Isatin-1,2,4-triazole hybrids |  | MIC: 8-500 µg/mL | Antibacterial(Gram-positive S. aureus, B. subtilis, and Gram-negative E. coli and P. aeruginosa) | [9] | |
| **5a** |  |  |  |  |  | |
| **5b** |  |  |  |  |  | |
| **5c** |  |  |  |  |  | |
| **5d** |  |  |  |  |  | |
| **5e** |  |  |  |  |  | |
| **5f** |  |  |  |  |  | |
| **5g** |  |  |  |  |  | |
| **5h** |  |  |  |  |  | |
| **6** | thiazolyl-isatin hybrids |  | IC50 2.05-252.56(μM) | Antiparasitic activity against forms of *T. cruzi* | [36] | |
| **6a** |  |  |  |  |  | |
| **6b** |  |  |  |  |  | |
| **6c** |  |  |  |  |  | |
| **6d** |  |  |  |  |  | |
| **6e** |  |  |  |  |  | |
| **6f** |  |  |  |  |  | |
| **6g** |  |  |  |  |  | |
| **6h** |  |  |  |  |  | |
| **6i** |  |  |  |  |  |  |
| **6 j** |  |  |  |  |  |  |
| **7** | Isatin -Thiazolidin-4-one Hybrids | **** |  | Anticonvulsant | [10] | |
| **7a** |  | **** |  |  |  | |
| **7b** |  | **** |  |  |  | |
| **7c** |  | **** |  |  |  | |
| **7d** |  | **** |  |  |  | |
| **7e** |  | **** |  |  |  | |
| **8** | Isatin–benzofuran hybrid | **** |  | Antibacterial  (E. faecalis,, E. faecium, E. coli, K. pneumoniae, P. aeruginosa, A. coacetious, E. cloacae, E. aerogenes, P. mirabilis, and C.  Freundii | [33] | |
| 8a |  | **** |  |  |  | |
| 8b |  | **** |  |  |  | |
| 8c |  | **** |  |  |  | |
| 8d |  | **** |  |  |  | |
| 8e |  | **** |  |  |  | |
| **9** | Benzofuran-isatin hybrids | **** | MIC: 0.25 and 0.5 µg/mL | Antitubercular (MTB H37Rv and MDR-TB strains) | [35] | |
| 9a |  | **** |  |  |  | |
| 9b |  | **** |  |  |  | |
| 9c |  | **** |  |  |  | |
| 9d |  | **** |  |  |  | |
| 10 | Isatin-thiophene hybrids | **** |  |  | [76] | |
| 10a |  | **** |  |  |  | |
| 10b |  | **** |  |  |  | |
| 11 | Isatin-thiophene hybrids | **** |  |  | [76] | |
| 11a |  | **** |  |  |  | |
| 11b |  | **** |  |  |  | |
| 11c |  | **** |  |  |  | |
| 11d |  | **** |  |  |  | |
| 11e |  | **** |  |  |  | |
| 11f |  | **** |  |  |  | |
| 12 | Indole-isatin hybrid | **** | 2.88-18.48 μM | Anticancer (HT-29, ZR-75, A-549 cell lines) | [41] | |
| 12a |  | **** |  |  |  | |
| 12b |  | **** |  |  |  | |
| 12c |  | **** |  |  |  | |
| 12d |  | **** |  |  |  | |
| 12e |  | **** |  |  |  | |
| 12f |  | **** |  |  |  | |
| 12g |  | **** |  |  |  | |
| 13 | Isatin-bisindole hybrids | **** |  |  |  | |
| 13a |  | **** |  |  |  | |
| 13b |  | **** |  |  |  | |
| 13c |  | **** |  |  |  | |
| 14 | 5-Flurorisatin-ciprofloxacin hybrids | **** | MIC: 7.81-125 µg/mL | Antibacterial (*S. aureus*, *S. epidermidis*, *M. luteus*, *E. coli*, *P. aeruginosa*, and *K. pneumonia)* | [9] | |
| 14a |  | **** |  |  |  | |
| 14b |  | **** |  |  |  | |
| 14c |  | **** |  |  |  | |
| 14d |  | **** |  |  |  | |
| 14e |  | **** |  |  |  | |
| 14f |  | **** |  |  |  | |
| 14g |  | **** |  |  |  | |
| 14h |  | **** |  |  |  | |
| 14i |  | **** |  |  |  | |
| 14j |  | **** |  |  |  | |
| 14k |  | **** |  |  |  | |
| **15** | Gatifloxacin-isatin hybrid | **** | 3.0 μg/mL | Anti-tuberculer MTB DNA gyrase | [17] | |
| **15a** |  | **** |  |  |  | |
| **15b** |  | **** |  |  |  | |
| **15c** |  | **** |  |  |  | |
| **15d** |  | **** |  |  |  | |
| **15e** |  | **** |  |  |  | |
| **15f** |  | **** |  |  |  | |
| **15g** |  | **** |  |  |  | |
| 16 | 5-(morpholinosulfonyl)isatin- thiazole hybrid | **** | MIC: 0.007-0.49 µg/mL | Antibacterial (*S. aureus*, *S. pyogenes*, *B. subtilis*, *Proteous vulgaris*/*P. vulgaris*, *Klebsiella pneumonia*/*K. pneumonia* and *Shigella flexneri*/*S. flexneri*,) | [9] | |
| 17 | 5-(morpholinosulfonyl)isatin- thiazole hybrid | **** | MIC: 0.03-3.9 *µ*g/mL | Antibacterial (*S. aureus*, *S. pyogenes*, *B. subtilis*, *Proteous vulgaris*/*P. vulgaris*, *Klebsiella pneumonia*/*K. pneumonia* and *Shigella flexneri*/*S. flexneri*,) | [9] | |
| 18 | Isatin-sulfonamide hybrid | **** | KIs of 8.9 and 9.2 nM | Anticancer(tumour-associated human carbonic anhydrase isoforms hCA IX and XII) | [2] | |
| 18a |  | **** |  |  |  | |
| 18b |  | **** |  |  |  | |
| 18c |  | **** |  |  |  | |
| 19 | Isatin-sulfonamide hybrids | **** | K1 from 7.8–32.6 nM | Anticancer(tumour-associated human carbonic anhydrase isoforms hCA I, II, IX, and XII) | **[**2] | |
| 19a |  | **** |  |  |  | |
| 19b |  | **** |  |  |  | |
| 19c |  | **** |  |  |  | |
| 19d |  | **** |  |  |  | |
| 19e |  | **** |  |  |  | |
| 19f |  | **** |  |  |  | |
| 19g |  | **** |  |  |  | |
| 19h |  | **** |  |  |  | |
| 20 | Isatin-sulfonamide hybrid |  | IC50 of 3.67 µM | antiproliferative activities (on HCT-116 cells) | [2] | |
| 20a |  |  |  |  |  | |
| 20b |  |  |  |  |  | |
| 20c |  |  |  |  |  | |
| 20d |  |  |  |  |  | |
| 20e |  |  |  |  |  | |
| 20f |  |  |  |  |  | |
| 21 | Isatin-sulfadimidine hybrids |  | EC_50_  27-50 µM | Antiviral(H1N1 inﬂuenza virus) | [44] | |
| 21a |  |  |  |  |  | |
| 21b |  |  |  |  |  | |
| 21c |  |  |  |  |  | |
| 21d |  |  |  |  |  | |
| 21e |  |  |  |  |  | |
| 22 | Isatin-pyridine hybrids |  | 14.45 -200 μM | Antiproliferative (HepG-2, A549 and MCF-7 cell lines.) | [45] | |
| 22a |  |  |  |  |  | |
| 22b |  |  |  |  |  | |
| 22c |  |  |  |  |  | |
| 23 | Isatin-pyridine hybrids |  | 2.5-19.3 μM | Antiproliferative (HepG-2, A549 and MCF-7 cell lines.) | [45] | |
| 24 | Isatin-pyridine hybrids |  | 6.3 to 85 μM | Antiproliferative (HepG-2, A549 and MCF-7 cell lines.) | [45] | |
| 24a |  |  |  |  |  | |
| 24b |  |  |  |  |  | |
| 24c |  |  |  |  |  | |
| 24d |  |  |  |  |  | |
| 25 | Isatin-pyridine oximes |  |  |  | [46] | |
| 25a |  |  | In vitro reactivation results  PXN (10 lM)b= 3 (± 0) | acetylcholinesterase reactivators |  | |
| 25b |  |  | In vitro reactivation results  PXN (10 lM)b= 8 (± 2) | acetylcholinesterase reactivators |  | |
| 25c |  |  | In vitro reactivation results  PXN (10 lM)b= 15 (± 1) | acetylcholinesterase reactivators |  | |
| 25d |  |  | In vitro reactivation results  PXN (10 lM)b= 14 (± 1) | acetylcholinesterase reactivators |  | |
| 25e |  |  | In vitro reactivation results  PXN (10 lM)b= 1 (± 0) | acetylcholinesterase reactivators |  | |
| 26 | Isatin-Schiff’s base and chalcone hybrids |  | IC50 μM/Ml  50*<,* 50*< and* 50*<* | anti-proliferative activities(MCF-7, HepG-2, and HCT-116) | [49] | |
| 27 | Isatin-Schiff’s base and chalcone hybrids |  | 2.81± 0.15, 2.88± 0.16 and 3.22± 0.21 μM/ML | anti-proliferative activities(MCF-7, HepG-2, and HCT-116) | [49] | |
| 28 | Isatin-Schiff’s base and chalcone hybrids |  | 8.76± 0.76 ,10.18± 0.84 and 13.10±0.95 μM/ML | anti-proliferative activities(MCF-7, HepG-2, and HCT-116) | [49] | |
| 29 | Isatin-quinazoline hybrids |  |  |  | [51] | |
| 29a |  |  | 7.4 ± 0.5 μM | antiproliferative activity  (MCF-7 cell line) |  | |
| 29b |  |  | 3.3 ± 0.4 μM | antiproliferative activity  (MCF-7 cell line) |  | |
| 29c |  |  | 9.8 ± 0.8 μM | antiproliferative activity  (MCF-7 cell line) |  | |
| 29d |  |  | 2.1 ± 0.19 μM | antiproliferative activity  (MCF-7 cell line) |  | |
| 29e |  |  | 5.5 ± 0.42 μM | antiproliferative activity  (MCF-7 cell line) |  | |
| 30 | Isatin-quinazoline hybrids |  |  |  | [50] | |
| 30a |  |  | IC50  13.18 ± 0.36 μM | anti-proliferative activity(MDA-MB-231 cell line) |  | |
| 30b |  |  | IC50  15.15 ± 0.15 μM | anti-proliferative activity(MDA-MB-231 cell line) |  | |
| 30c |  |  | IC50  17.93 ± 0.11 μM | anti-proliferative activity(MDA-MB-231 cell line) |  | |
| 30d |  |  | IC50  19.68 ± 0.26 μM | anti-proliferative activity(MDA-MB-231 cell line) |  | |
| 31 | Isatin-pthalazine hybrids |  |  |  | [50] | |
| 31a |  |  | 13.96 ± 0.10 μM | anti-proliferative activity  (MDA-MB-231 cell line) |  | |
| 31b |  |  | 15.62 ± 0.14 μM | anti-proliferative activity  (MDA-MB-231 cell line) |  | |
| 31c |  |  | IC50  17.63 ± 0.37 μM | anti-proliferative activity (MDA-MB-231 cell line) |  | |
| 31d |  |  | IC50  21.39 ± 0.13 μM | anti-proliferative activity  (MDA-MB-231 cell line) |  | |
| 31e |  |  | IC50  12.86 ± 0.12 μM | anti-proliferative activity  (MDA-MB-231 cell line) |  | |
| 31f |  |  | IC50  12.94 ± 0.23 μM | anti-proliferative activity  (MDA-MB-231 cell line) |  | |
| 32 | Isatin–carbohydrazide hybrids |  | MIC: 3.9 –83.3 μg/ml | Antibacterial (S. aureus, B. subtilis, E. coli, and P. aeruginosa) | [33] | |
| 32a |  |  |  |  |  | |
| 32b |  |  |  |  |  | |
| 33 | Isatin-nicotinohydrazide hybrids |  | MIC 3.9 mg/mL | Antitubacular  (MTB H37Rv and MDR-TB) | [35] | |
| 33a |  |  |  |  |  | |
| 33b |  |  |  |  |  | |
| 34 | Isatin–nicotinohydrazide hybrids |  | MIC:  0.24- 7.81 mg/mL. | Antibacteria  (Mycobacterium tuberculosis (ATCC 27294)) | [48] | |
| 34a |  |  |  |  |  | |
| 34b |  |  |  |  |  | |
| 34c |  |  |  |  |  | |
| 34d |  |  |  |  |  | |
| 34e |  |  |  |  |  | |
| 34f |  |  |  |  |  | |
| 34g |  |  |  |  |  | |
| 34h |  |  |  |  |  | |
| 35 | Isatin-carbohydrazide hybrids |  | MIC: 12.5-100 *µ*g/mL | Antibacterial  (*Salmonella typhi*, *E. coli*, *Vibrio cholera*, *S. aureus*, *S. epidermidis*, *K. pneumonia*, *P. aeruginosa*, *S. flexnari* and *Citrobactor ferundi*.) | [9] | |
| 35a |  |  |  |  |  | |
| 35b |  |  |  |  |  | |
| 35c |  |  |  |  |  | |
| 35d |  |  |  |  |  | |
| 35e |  |  |  |  |  | |
| 35f |  |  |  |  |  | |
| 35g |  |  |  |  |  | |
| 36 | Isatin-*β*-thiosemicarbazone hybrids |  | (MIC: 0.39-6.25 *µ*g/mL) | Antibacterial  Gram-positive( *E. coli* and *S. dysenterie*,) | [9] | |
| 36a |  |  |  |  |  | |
| 36b |  |  |  |  |  | |
| 36c |  |  |  |  |  | |
| 36d |  |  |  |  |  | |
| 36e |  |  |  |  |  | |
| 36f |  |  |  |  |  | |
| 36g |  |  |  |  |  | |
| 36h |  |  |  |  |  | |
| 36i |  |  |  |  |  | |
| 37 | Isatin-thiosemicarbazone hybrids |  | MIC 3.12-100 mM | Atifungal  (Aspergillus niger) | [20] | |
| 37a |  |  |  |  |  | |
| 37b |  |  |  |  |  | |
| 37c |  |  |  |  |  | |
| 37d |  |  |  |  |  | |
| 37e |  |  |  |  |  | |
| 37f |  |  |  |  |  | |
| 38 | Isatin β-thiosemicarbazone hybrid |  | EC_50_  3.40 µM | Anti-HIV | [15] | |
| 39 | Isatin β-thiosemicarbazone hybrids |  | EC_50_  2.62 µM | Anti-HIV | [15] | |
| 40 | Isatin β-thiosemicarbazone hybrid |  | EC_50_ 3.12 µM | Anti-HIV | [15] | |
| 41 | Isatin-oxime hybrid |  | CC_50_ :7.46-190.8 μM | Antiviral (respiratory syncytial virus (RSV) | [52] | |
| 41a |  |  |  |  |  | |
| 41b |  |  |  |  |  | |
| 41c |  |  |  |  |  | |
| 41d |  |  |  |  |  | |
| 41e |  |  |  |  |  | |
| 41f |  |  |  |  |  | |
| 41g |  |  |  |  |  | |
| 41m |  |  |  |  |  | |
| 42 | Isatine-nitrone hybrid |  | CC_50_: 68.7-105.7 μM | Antiviral (respiratory syncytial virus (RSV) | [52] | |
| 42a |  |  |  |  |  | |
| 42b |  |  |  |  |  | |
| 42c |  |  |  |  |  | |
| 43 | Piperazine-isatin hybrid |  | Docking results= S (kcal/mol)=  -6.8147421 | Antiviral  (SARS-CoV-2 protease enzyme) | [54] | |
| 44 | Uracil-isatin hybrids |  |  |  | [55] | |
| 44a |  |  | >100 **µM** | Anticancer  (HeLa cancer cell lines) |  | |
| 44b |  |  | >100 **µM** | Anticancer  (HeLa cancer cell lines) |  | |
| 44c |  |  | >100 **µM** | Anticancer  (HeLa cancer cell lines) |  | |
| 44d |  |  | 52.57 **µM** | Anticancer  (HeLa cancer cell lines) |  | |
| 44e |  |  | >100 **µM** | Anticancer  (HeLa cancer cell lines) |  | |
| 44f |  |  | >100 **µM** | Anticancer  (HeLa cancer cell lines) |  | |
| 45 | Coumarin-isatin hybrids |  |  |  | [56] | |
| 45a |  |  | 3.73 [3.4–4.0] and 4.13 [3.7–4.5] μmol/L | antileishmanial  *L. tropica* promastigote and axenic amastigote forms. |  | |
| 45b |  |  | 1.62 [1.4–1.8] and 2.85 [2.6–3.0] μmol/L | antileishmanial  *L. tropica* promastigote and axenic amastigote forms. |  | |
| 45c |  |  | 0.10 [0.4–0.17] and 0.87 [0.8–0.9] μmol/L | antileishmanial  *L. tropica* promastigote and axenic amastigote forms. |  | |
| 46 | Isatin-coumarin hybrids |  |  |  | [122] | |
| 46a |  |  | 39.46 μM | Anticancer  HepG2 |  | |
| 46b |  |  | 29.67 μM | Anticancer  HepG2 |  | |
| 46c |  |  | >50 μM | Anticancer  HepG2 |  | |
| 46d |  |  | *>*50 μM | Anticancer  HepG2 |  | |
| 46e |  |  | 21.47 μM | Anticancer  HepG2 |  | |
| 46f |  |  | 19.89 μM | Anticancer  HepG2 |  | |
| 46g |  |  | 49.42 μM | Anticancer  HepG2 |  | |
| 46h |  |  | 44.79 μM | Anticancer  HepG2 |  | |
| 46i |  |  | 46.77 μM | Anticancer  HepG2 |  | |
| 46j |  |  | 39.93 μM | Anticancer  HepG2 |  | |
| 46k |  |  | *>*50 μM | Anticancer  HepG2 |  | |
| 46l |  |  | *>*50 μM | Anticancer  HepG2 |  | |
| 47 | Isatin-coumarin hybrid |  |  |  | [123] | |
| 47a |  |  | MIC :  >32 μg/mL | Antitubercular |  | |
| 47b |  |  | MIC : >32 μg/mL | Antitubercular |  | |
| 47c |  |  | MIC : >32 μg/mL | Antitubercular |  | |
| 47d |  |  | MIC : >32 μg/mL | Antitubercular |  | |
| 48 | Isatin-thiolactone hybrids |  |  |  | [57] | |
| 48a |  |  | 31.70 µM | Antiplasmodial activity  FP-2a |  | |
| 48b |  |  | 47.80 µM | Antiplasmodial activity  FP-2a |  | |
| 48c |  |  | 89.60 µM | Antiplasmodial activity  FP-2a |  | |
| 48d |  |  | 17.80 µM | Antiplasmodial activity  FP-2a |  | |
| 48e |  |  | >100 µM | Antiplasmodial activity  FP-2a |  | |
| 48f |  |  | 40.20 µM | Antiplasmodial activity  FP-2a |  | |
| 49 | Dihydropyrimidinone-Isatin hybrids |  | 64->100 nM | Antiviral(HIV-1 Reverse Transcriptase Inhibitors) | [47] | |
| 49a |  |  |  |  |  | |
| 49b |  |  |  |  |  | |
| 49c |  |  |  |  |  | |
| 49d |  |  |  |  |  | |
| 49e |  |  |  |  |  | |
| 50 | Isatin-thiazole hybrids |  | MIC: 77.16 μmol/L | antimycobacterial | [17] | |
| 51 | Isatin pyrimidine hybrids |  | MIC: 62.5 μg/mL | Antimycobacterial MTB H_37_Rv | [17] | |
| 52 | Isatin pyrimidine hybrids |  | MIC: 3.10 μg/ML | Antimycobacterial MTB H_37_Rv | [17] | |
| 53 | Isatin pyrimidine hybrids |  | MIC: 3.12 μg/mL. | Antimycobacterial MTB H_37_Rv | [17] | |
| 54 | Isatin-quinoline hybrid |  | 118 nM | Anti-malaria | [27] | |
| 55 | Isatin-quinoline hybrid |  | 128 nM | Anti-malaria | [27] | |
| 56 | Thioacetazone-isatin hybrid |  | GI: 90–96% at 6.25 mg/ML | Antitubercular MTB H37 Rv | [17] | |
| 57 | Thioacetazone-isatin hybrid |  | GI: 90–96% at 6.25 mg/ML | Antitubercular MTB H37 Rv | [17] | |
| 58 | Thioacetazone-isatin hybrid |  | GI: 90–96% at 6.25 mg/ML | Antitubercular MTB H37 Rv | [17] | |
| 59 | Thioacetazone-isatin hybrid |  | GI: 90–96% at 6.25 mg/ML | Antitubercular MTB H37 Rv | [17] | |
| 60 | Isatin-imine hybrids |  | MIC: 10-30  µg/mL | Antibacterial(*Salmonella typhi*, *E. coli*, *Vibrio cholera*, *S. aureus*, *S. epidermidis*, *K. pneumonia*, *P. aeruginosa*, *S. flexnari* and *Citrobactor ferundi*.) | [9] | |
| 60a |  |  |  |  |  | |
| 60b |  |  |  |  |  | |
| 60c |  |  |  |  |  | |
| 61 | Bis-isatin derivatives |  |  |  | [131] | |
| 61a |  |  | MIC :  100 and 64 μg/mL | Anti-mycobacterial activities  MTB H37Rv and MDR-TB |  | |
| 61b |  |  | MIC: 100 and 128 μg/mL | Anti-mycobacterial activities  MTB H37Rv and MDR-TB |  | |
| 61c |  |  | MIC :  100 and 32 μg/mL | Anti-mycobacterial activities  MTB H37Rv and MDR-TB |  | |
| 61d |  |  | MIC :  100 and 64 μg/mL | Anti-mycobacterial activities  MTB H37Rv and MDR-TB |  | |
| 61e |  |  | MIC :  100 and 128 μg/mL | Anti-mycobacterial activities  MTB H37Rv and MDR-TB |  | |
| 61f |  |  | MIC :  200 and 256 μg/mL | Anti-mycobacterial activities  MTB H37Rv and MDR-TB |  | |
| 61g |  |  | MIC :  100 and 256 μg/mL | Anti-mycobacterial activities  MTB H37Rv and MDR-TB |  | |
| 61h |  |  | MIC: 200 and 256 μg/mL | Anti-mycobacterial activities  MTB H37Rv and MDR-TB |  | |
| 61i |  |  | MIC :  25 and 16 μg/mL | Anti-mycobacterial activities  MTB H37Rv and MDR-TB |  | |
| 62 | Substituted isatin derivatives |  |  |  | [132] | |
| 62a |  |  | 0.25± 0.14 μM | Anti-tumor activity(human tumor cell Jurkat) |  | |
| 62b |  |  | 0.20± 0.03 μM | Anti-tumor activity(human tumor cell Jurkat) |  | |
| 62c |  |  | 0.07± 0.04 μM | Anti-tumor activity(human tumor cell Jurkat) |  | |
| 62d |  |  | 0.38± 0.09 μM | Anti-tumor activity(human tumor cell Jurkat) |  | |
| 62e |  |  | 0.08± 0.01 μM | Anti-tumor activity(human tumor cell Jurkat) |  | |
| 62f |  |  | 0.36± 0.09 μM | Anti-tumor activity(human tumor cell Jurkat) |  | |
| 62g |  |  | 0.05± 0.02 μM | Anti-tumor activity(human tumor cell Jurkat) |  | |
| 63 | Substituted isatin derivatives |  |  | Anti-tumor activity(human tumor cell Jurkat) | [132] | |
| 63a |  |  | 0.03± 0.001 μM | Anti-tumor activity(human tumor cell Jurkat) |  | |
| 63b |  |  | 0.79± 0.09 μM | Anti-tumor activity(human tumor cell Jurkat) |  | |
| 63c |  |  | 3.11± 1.52 μM | Anti-tumor activity(human tumor cell Jurkat) |  | |
| 63d |  |  | >10 μM | Anti-tumor activity(human tumor cell Jurkat) |  | |
| 64 | α,β-unsaturatedketone (Micheal accepter)-isatin hybrids |  |  |  | [53] | |
| 64a |  |  | 29.9±2.7, 14.4 ±1.5 and 20.4±1.3 μM | Anticancer  (BGC-823 cells, SGC-7901 cells and  NCI-H460 cells) |  | |
| 64b |  |  | 19.8 ±1.2 ,23.2±2.0 and 5.5±0.3 μM | Anticancer  (BGC-823 cells, SGC-7901 cells and  NCI-H460 cells) |  | |
| 64c |  |  | 6.9±0.4 ,8.5±0.9 and 6.5±0.7 μM | Anticancer  (BGC-823 cells, SGC-7901 cells and  NCI-H460 cells) |  | |
| 64d |  |  | 9.2±0.8, 11.5±0.3 and 5.4±0.3 μM | Anticancer  (BGC-823 cells, SGC-7901 cells and  NCI-H460 cells) |  | |
| 64e |  |  | 11.8±0.7, 17.4±0.0 and 8.5±0.3 μM | Anticancer  (BGC-823 cells, SGC-7901 cells and  NCI-H460 cells) |  | |
| 64f |  |  | 9.1±1.4, 12.8±0.4 and 7.7±0.6 μM | Anticancer  (BGC-823 cells, SGC-7901 cells and  NCI-H460 cells) |  | |
| 64g |  |  | 11.1±2.3 ,12.4±1.2 and 7.5±1.4 μM | Anticancer  (BGC-823 cells, SGC-7901 cells and  NCI-H460 cells) |  | |
| 64h |  |  | 53.7 ±1.7 ,56.3±6.1and 61.6±1.6 μM | Anticancer  (BGC-823 cells, SGC-7901 cells and  NCI-H460 cells) |  | |
| 64i |  |  | 9.5±0.1,13.1±0.1 and 11.0±0.9 μM | Anticancer  (BGC-823 cells, SGC-7901 cells and  NCI-H460 cells) |  | |
| 64j |  |  | 7.7±0.3 ,8.4±0.6 and 9.8±0.7 μM | Anticancer  (BGC-823 cells, SGC-7901 cells and  NCI-H460 cells) |  | |
| 64k |  |  | 13.2±1.3, 17.8± 1.2 and 13.7±1.3 μM | Anticancer  (BGC-823 cells, SGC-7901 cells and  NCI-H460 cells) |  | |
| 64l |  |  | 9.0 ±0.9 ,10.2±1.0 and 11.5±3.7 μM | Anticancer  (BGC-823 cells, SGC-7901 cells and  NCI-H460 cells) |  | |
| 64m |  |  | 14.3±1.2 ,18.2±1.3 and 18.4±1.4 μM | Anticancer  (BGC-823 cells, SGC-7901 cells and  NCI-H460 cells) |  | |
| 64n |  |  | 13.9±0.9 ,18.3±0.6 and 10.9±0.3 μM | Anticancer  (BGC-823 cells, SGC-7901 cells and  NCI-H460 cells) |  | |
| 64o |  |  | 15.7±1.9 ,17.6±0.3 and 13.3±1.7 μM | Anticancer  (BGC-823 cells, SGC-7901 cells and  NCI-H460 cells) |  | |
| 64p |  |  | 10.2±1.3 ,16.3±0.7 and 10.2±2.0 μM | Anticancer  (BGC-823 cells, SGC-7901 cells and  NCI-H460 cells) |  | |
| 65 | α,β-unsaturatedketone (Micheal accepter)-isatin hybrids |  |  |  | [53] | |
| 65a |  |  | 3.6 ±0.6 ,5.7±1.2 and 3.2±0.7 µM | Anticancer  (BGC-823 cells, SGC-7901 cells and  NCI-H460 cells) |  | |
| 65b |  |  | 6.2±1.0 ,8.6±0.6 and 9.7±0.7 µM | Anticancer  (BGC-823 cells, SGC-7901 cells and  NCI-H460 cells) |  | |
| 65c |  |  | 7.5±0.9 ,11.5±0.7 and 10.3±0.6 µM | Anticancer  (BGC-823 cells, SGC-7901 cells and  NCI-H460 cells) |  | |
| 65d |  |  | 9.1±1.5 ,14.1 ±0.3 and 12.3±1 µM | Anticancer  (BGC-823 cells, SGC-7901 cells and  NCI-H460 cells) |  | |
| 66 | Isatin–1,2,3‐triazole hybrids |  | 5.7 to >50 μM | Anticancer (prostrate,breast, lung and cervical cancer cells) | [8] | |
| 66a |  |  |  |  |  | |
| 66b |  |  |  |  |  | |
| 66c |  |  |  |  |  | |
| 66d |  |  |  |  |  | |
| 66e |  |  |  |  |  | |
| 66f |  |  |  |  |  | |
| 66g |  |  |  |  |  | |
| 66h |  |  |  |  |  | |
| 67 | Isatin–1,2,3‐triazole hybrids |  | 3.7 to >50 μM | Anticancer (prostrate,breast, lung and cervical cancer cells) | [8] | |
| 67a |  |  |  |  |  | |
| 67b |  |  |  |  |  | |
| 67c |  |  |  |  |  | |
| 67d |  |  |  |  |  | |
| 67e |  |  |  |  |  | |
| 67f |  |  |  |  |  | |
| 67g |  |  |  |  |  | |
| 68 | Isatin–1,2,3‐triazole hybrids |  |  |  | [8] | |
| 68a |  |  |  |  |  | |
| 68b |  |  |  |  |  | |
| 68c |  |  |  |  |  | |
| 68d |  |  |  |  |  | |
| 68e |  |  |  |  |  | |
| 68f |  |  |  |  |  | |
| 69 | Isatin–1,2,3‐triazole hybrids |  | 1.54μM | Anticancer(prostrate,breast, lung and cervical cancer cells) | [8] | |
| 70 | Isatin–thiazole/thiazolidinone hybrids |  |  |  | [8] | |
| 70a |  |  |  |  |  | |
| 70b |  |  |  |  |  | |
| 70c |  |  |  |  |  | |
| 70d |  |  |  |  |  | |
| 70e |  |  |  |  |  | |
| 71 | Isatin–thiazole/thiazolidinone hybrids |  | 1.87–24.59 μM | Anticancer (lung, breast,colon cancer cells) | [8] | |
| 71a |  |  |  |  |  | |
| 71b |  |  |  |  |  | |
| 72 | Isatin–thiazole/thiazolidinone hybrids |  |  |  | [8] | |
| 72a |  |  |  |  |  | |
| 73 | Isatin–thiazole/thiazolidinone hybrids |  | >10 μM | Anticancer (lung, breast, colon cancer cells) | [8] | |
| 74 | Isatin–thiazole/thiazolidinone hybrids |  | 7.6–152.1 μM; | Anticancer (lung, breast, colon cancer cells) | [8] | |
| 75 | Isatin–thiazole/thiazolidinone hybrids |  | 13.2–127.3 μM | Anticancer (lung, breast, colon cancer cells) | [8] | |
| 76 | Isatin–thiazole/thiazolidinone hybrids |  | >50 μM; | Anticancer (lung, breast, colon cancer cells) | [8] | |
| 77 | Isatin–thiazole/thiazolidinone hybrids |  | 55.39–72.67 μM | Anticancer (lung, breast, colon cancer cells) | [8] | |
| 78 | Isatin–pyrazole |  | 30.41 and 29.69 | Anticancer (lung, breast, colon cancer cells) | [8] | |
| 78a |  |  |  |  |  | |
| 78b |  |  |  |  |  | |
| 79 | Isatin–pyrazole |  |  |  | [8] | |
| 79a |  |  | 58.48 μM | Anticancer (breast cancer cells) |  | |
| 80 | Isatin–thiazolidinone–pyrazoline hybrid |  | GI50: 0.046–0.80 μM | Anticancer(lung, breast, colon cancer cells) | [8] | |
| 80a |  |  |  |  |  | |
| 80b |  |  |  |  |  | |
| 80c |  |  |  |  |  | |
| 80d |  |  |  |  |  | |
| 80e |  |  |  |  |  | |
| 80f |  |  |  |  |  | |
| 81 | Isatin-pyrazoline hybrid |  | 1.06–54.35 μM | Anticancer (lung, breast, colon cancer cells) | [8] | |
| 81a |  |  |  |  |  | |
| 81b |  |  |  |  |  | |
| 81c |  |  |  |  |  | |
| 81d |  |  |  |  |  | |
| 82 | Spiro‐isatin–pyrazoline hybrids |  | GI50: 1.30–6.25 μM | Anticancer (lung, breast, colon cancer cells) | [8] | |
| 82a |  |  |  |  |  | |
| 82b |  |  |  |  |  | |
| 83 | Isatin–pyrazolone hybrid |  | 78.76 to >1,000 μM | Anticancer (lung, breast, colon cancer cells) | [8] | |
| 83a |  |  |  |  |  | |
| 83b |  |  |  |  |  | |
| 83c |  |  |  |  |  | |
| 83d |  |  |  |  |  | |
| 84 | Isatin–benzimidazole hybrids |  | 22.59–64.14 nM | Anticancer (lung, breast, colon cancer cells) | [8] | |
| 84a |  |  |  |  |  | |
| 84b |  |  |  |  |  | |
| 84c |  |  |  |  |  | |
| 84d |  |  |  |  |  | |
| 85 | Isatin-imidazolone hybrid |  | 3.8–87.8 μM | Anticancer (lung, breast, colon cancer cells) | [8] | |
| 85a |  |  |  |  |  | |
| 85b |  |  |  |  |  | |
| 86 | Isatin-imidazolone hybrid |  | 18.5–311 μM | Anticancer (lung, breast, colon cancer cells) | [8] | |
| 87 | Isatin–tetrazole hybrids |  |  |  |  | |
| 87a |  |  |  |  |  | |
| 87b |  |  |  |  |  | |
| 88 | Isatin–1,3,4‐thiadiazole hybrid |  | 0.65–17.09 μM | Anticancer (lung, breast, colon cancer cells) | [8] | |
| 88a |  |  |  |  |  | |
| 88b |  |  |  |  |  | |
| 89 | Isatin–1,3,4‐thiadiazole hybrid |  | 10.46–21.41 μM | Anticancer (lung, breast, colon cancer cells) | [8] | |
| 89a |  |  |  |  |  | |
| 89b |  |  |  |  |  | |
| 89c |  |  |  |  |  | |
| 89d |  |  |  |  |  | |
| 89e |  |  |  |  |  | |
| 90 | Isatin–1,3,4‐thiadiazole hybrid |  | 1.61–25.46 μM | Anticancer (lung, breast, colon cancer cells) | [8] | |
| 90a |  |  |  |  |  | |
| 90b |  |  |  |  |  | |
| 90c |  |  |  |  |  | |
| 91 | Isatin–thiazolidinone–thiazole hybrid |  | MIC: 0.49–3.9 μg/mL | Antibacterial (S. aureus, P. aeruginosa, and E. coli,) | [33] | |
| 91a |  |  |  |  |  | |
| 91b |  |  |  |  |  | |
| 91c |  |  |  |  |  | |
| 92 | Isatin–furan hybrid |  | 8.5–14 mm at 20 mg/mL | Antibacterial (E. coli, P. aeruginosa, S. aureus, and B. subtilis) | [33] | |
| 92a |  |  |  |  |  | |
| 92b |  |  |  |  |  | |
| 92c |  |  |  |  |  | |
| 93 | Isatin-Naphthalene hybrid |  | 0.37 μM | Antiviral(SARS-CoV1) | [14] | |
| 94 | Isatin-oxidiazole hybrids |  | Ki: 9.10 nM | Antiviral(SARS-Co-V-2 Mpro) | [14] | |
| 95 | Isatin-oxidiazole hybrids |  | Ki: 11.33 nM | Antiviral(SARS-Co-V-2 Mpro) | [14] | |
| 96 | Isatin-imidazole hybrids |  | KI: 0.317 μM | Antiviral(SARS-CoV-2 RNAdependent RNA polymerase (RdRp)) | [34] | |
| 97 | Isatin-imidazole hybrids |  | KI: 0.348 μM | Antiviral(SARS-CoV-2 RNAdependent RNA polymerase (RdRp)) | [34] | |
| 98 | Isatin-imidazole hybrids |  | KI: 0.188 μM | Antiviral(SARS-CoV-2 RNAdependent RNA polymerase (RdRp)) | [34] | |
| 99 | Isatin-imidazole hybrids |  | KI: 0.492 μM | Antiviral(SARS-CoV-2 RNAdependent RNA polymerase (RdRp)) | [34] | |
| 100 | Isatin-imidazole hybrids |  | KI: 0.397 μM | Antiviral(SARS-CoV-2 RNAdependent RNA polymerase (RdRp)) | [34] | |
| 101 | Isatin-imidazole hybrids |  | KI: 0.332 μM | Antiviral(SARS-CoV-2 RNAdependent RNA polymerase (RdRp)) | [34] | |
| 102 | Isatin-imidazole hybrids |  | KI: 0.294 μM | Antiviral(SARS-CoV-2 RNAdependent RNA polymerase (RdRp)) | [34] | |
| 103 | Isatin-imidazole hybrids |  | KI: 0.102 μM | Antiviral(SARS-CoV-2 RNAdependent RNA polymerase (RdRp)) | [34] | |
| 104 | Isatin-imidazole hybrids |  | KI: 0.206 μM | Antiviral(SARS-CoV-2 RNAdependent RNA polymerase (RdRp)) | [34] | |
| 105 | Isatin-imidazole hybrids |  | KI: 0.133 μM | Antiviral(SARS-CoV-2 RNAdependent RNA polymerase (RdRp)) | [34] | |
| 106 | benzofuran-isatin-hydroxylimine/- thiosemicarbazide hybrids |  | MIC: 0.22 and 0.86 μg/mL, | Antitubercular (MTB H37Rv and MDR-TB) | [35] | |
| 107 | isatin-propylene-1H-1,2,3-triazole-4-methylenemoxifloxacin hybrids |  | 2.0 µg/mL | Antitubercular (H37Rv) | [35] | |
| 108 | Isatin-2,3-dihydrooxazole hybrids |  | MIC and minimum bactericidal concentration (MBC) values ranging from 25 to 100 µg/mL | Antibacterial (Gram-positive *B. subtilis*, Gram-negative *Enterobacter* and *K. pneumonia*,) | [9] | |
| 108a |  |  |  |  |  | |
| 108b |  |  |  |  |  | |
| 108c |  |  |  |  |  | |
| 108d |  |  |  |  |  | |
| 108e |  |  |  |  |  | |
| 108f |  |  |  |  |  | |
| 108g |  |  |  |  |  | |
| 109 | Isatin-2,3-dihydrooxazole hybrids |  | MIC and minimum bactericidal concentration (MBC) values ranging from 25 to 100 *µ*g/mL | Antibacterial (Gram-positive *B. subtilis*, Gram-negative *Enterobacter* and *K. pneumonia*,) | [9] | |
| 109a |  |  |  |  |  | |
| 109b |  |  |  |  |  | |
| 109c |  |  |  |  |  | |
| 109d |  |  |  |  |  | |
| 109e |  |  |  |  |  | |
| 109f |  |  |  |  |  | |
| 109g |  |  |  |  |  | |
| 110 | Isatin-2,3-dihydrooxazole hybrids |  | MIC: 1-15 µg/mL | Antibacterial (Gram-positive *S. aureus* and *B. cereus*, and Gram-negative *E. coli* and *S. dysenterie*,) | [9] | |
| 110a |  |  |  |  |  | |
| 110b |  |  |  |  |  | |
| 110c |  |  |  |  |  | |
| 110d |  |  |  |  |  | |
| 111 | Azide-containing isatin-pyrazoline hybrids |  | MIC: 6.25-50 *µ*g/mL | Antibacterial (*S. aureus*, *B. subtilis*, *E. coli* and *P. aeruginosa*,) | [9] | |
| 111a |  |  |  |  |  | |
| 111b |  |  |  |  |  | |
| 111c |  |  |  |  |  | |
| 111d |  |  |  |  |  | |
| 111e |  |  |  |  |  | |
| 112 | Isatin-1,3,4-thiadiazole hybrid |  | MIC: 62.5-500 µg/mL | Antibacterial (Gram-positive *S. aureus* and *B. cereus*, and Gram-negative *E. coli* and *S. dysenterie*,) | [9] | |
| 112a |  |  |  |  |  | |
| 112b |  |  |  |  |  | |
| 112c |  |  |  |  |  | |
| 112d |  |  |  |  |  | |
| 112e |  |  |  |  |  | |
| 112f |  |  |  |  |  | |
| 112g |  |  |  |  |  | |
| 112h |  |  |  |  |  | |
| 112i |  |  |  |  |  | |
| 112j |  |  |  |  |  | |
| 112k |  |  |  |  |  | |
| 112l |  |  |  |  |  | |
| 113 | Isatin-Ciprofloxacin-/lomefloxacin hybrids |  |  |  | [9] | |
| 113a |  |  |  |  |  | |
| 113b |  |  |  |  |  | |
| 113c |  |  |  |  |  | |
| 113d |  |  |  |  |  | |
| 114 | Isatin-fluoroquinolone hybrids |  | MIC: 0.00003-0.0290µg/mL | Antibacterial (*Salmonella typhi*, *E. coli*, *Vibrio cholera*, *S. aureus*, *S. epidermidis*, *K. pneumonia*, *P. aeruginosa*, *S. flexnari* and *Citrobactor ferundi*.) | [9] | |
| 115 | Isatin-furan hybrids |  | inhibition zone ranging from 8.5 to 14 mm at 20mg/mL | Antibacterial ( *E. coli*, *P. aeruginosa*, *S. aureus* and *B. Subtilis*,) | [9] | |
| 116 | Carbohydrazone tethered isatin dimers |  | MIC: 6.25-100  µg/mL | Antibacterial (E. coli, P. aeruginosa, S. aureus and B.  Subtilis) | [9] | |
| 116a |  |  |  |  |  | |
| 116b |  |  |  |  |  | |
| 116c |  |  |  |  |  | |
| 116d |  |  |  |  |  | |
| 116e |  |  |  |  |  | |
| 116f |  |  |  |  |  | |
| 116g |  |  |  |  |  | |
| 116h |  |  |  |  |  | |
| 116i |  |  |  |  |  | |
| 116j |  |  |  |  |  | |
| 117 | Isatin-benzene sulfonamide hybrids |  | Ki: 0.47 to 2.83 nM | Anticancer (tumour-associated human carbonic anhydrase isoforms (hCA XII) | [2] | |
| 117a |  |  |  |  |  | |
| 117b |  |  |  |  |  | |
| 118 | Ciprofloxacin-/lomefloxacin-isatin hybrids |  | MIC: 0.10 - 2.50 *µ*g/mL | Antibacterial (*Salmonella typhi*, *E. coli*, *Vibrio cholera*, *S. aureus*, *S. epidermidis*, *Klebsiella pneumonia* (*K. pneumonia*), *P. aeruginosa*, *Shigella flexnari* and *Citrobactor ferundi* ) | [42] | |
| 118a |  |  |  |  |  | |
| 118b |  |  |  |  |  | |
| 118c |  |  |  |  |  | |
| 118d |  |  |  |  |  | |
| 119 | Isatin-benzene sulfonamide hybrid |  |  | Antifungal (Inhibition of carbonic anhydrases from pathogenic Candida species, CgNce103 from C. glabrata) | [21] | |
| 119a |  |  | KI:  136 nM |  |  | |
| 119b |  |  | KI:  139 nM |  |  | |
| 119c |  |  | KI :  91.5 nM |  |  | |
| 119d |  |  | KI:  65.7 nM |  |  | |
| 119e |  |  | KI:  295 nM |  |  | |
| 120 | Isatin-3-pyridylamine hybrid |  | MIC:  3.9 mg/mL. | Antibacteria  (Mycobacterium tuberculosis (ATCC 27294)) | [48] | |
| 121 | Aza-isatin oxime hybrid |  | CC_50_ 12.4-223 μM | Antiviral (respiratory syncytial virus (RSV) | [52] | |
| 121a |  |  |  |  |  | |
| 121b |  |  |  |  |  | |
| 121c |  |  |  |  |  | |
| 121d |  |  |  |  |  | |
| 122 | Isatin-benzo[b]thiophene hybrid |  | IC_50_: 0.95 μM | Antiviral  SARS-CoV main protease.( 3CLpro) | [40] | |
| 123 | Isatin-azole hybrids |  | MIC:  64-256 µg/mL | Antibacterial gram-positive bacteria *S. aureus* | [23] | |
| 123a |  |  |  |  |  | |
| 123b |  |  |  |  |  | |
| 123c |  |  |  |  |  | |
| 123d |  |  |  |  |  | |
| 124 | Isatin-azole hybrids |  | MIC :  64-256 µg/mL | Antibacterial gram-positive bacteria *S. aureus* | [23] | |
| 124a |  |  |  |  |  | |
| 125 | Isatin 1,2,4-triazole hybrid |  | MIC : 128-256 µg/mL | Antibacterial gram-positive bacteria *S. aureus* | [23] | |
| 125a |  |  |  |  |  | |
| 125b |  |  |  |  |  | |
| 125c |  |  |  |  |  | |
| 125d |  |  |  |  |  | |
| 125e |  |  |  |  |  | |
| 126 | Isatin 1,2,4-triazole hybrid |  | MIC 64-512  µg/mL | Antibacterial gram-positive bacteria *S. aureus* | [23] | |
| 126a |  |  |  |  |  | |
| 126b |  |  |  |  |  | |
| 126c |  |  |  |  |  | |
| 126d |  |  |  |  |  | |
| 127 | Isatin 1,2,4-triazole hybrid |  | MIC: 128 µg/mL | Antibacterial gram-positive bacteria *S. aureus* | [23] | |
| 128 | Isatin-triazole hydrazones. hybrids |  |  |  | [37] | |
| 128a |  |  | >20 µM | Anticancer MARK4 inhibition |  | |
| 128b |  |  | 14.10 ± 0.20 µM | Anticancer MARK4 inhibition |  | |
| 128c |  |  | 18.66 ± 1.22 µM | Anticancer MARK4 inhibition |  | |
| 128d |  |  | 12.45 ± 0.64 µM | Anticancer MARK4 inhibition |  | |
| 128e |  |  | 19.24 ± 1.45 µM | Anticancer MARK4 inhibition |  | |
| 128f |  |  | >20 µM | Anticancer MARK4 inhibition |  | |
| 128g |  |  | 1.54 ± 0.22 µM | Anticancer MARK4 inhibition |  | |
| 128h |  |  | 8.40 ± 0.12 µM | Anticancer MARK4 inhibition |  | |
| 128i |  |  | 10.22 ± 0.44 µM | Anticancer MARK4 inhibition |  | |
| 129 | 1H‐123‐ triazole‐tethered moxifloxacin ‒isatin hybrids |  |  |  | [38] | |
| 129a |  |  | MIC :  0.20 and 0.25 μg/mL | anti‐mycobacterial activity  MTB H37Rv and MDR– MTBa |  | |
| 129b |  |  | MIC :  0.78 and 0.25 μg/mL | anti‐mycobacterial activity  MTB H37Rv and MDR– MTBa |  | |
| 129c |  |  | MIC :  0.025and 0.06 μg/mL | anti‐mycobacterial activity  MTB H37Rv and MDR– MTBa |  | |
| 129d |  |  | MIC :  0.10 and 0.12 μg/mL | anti‐mycobacterial activity  MTB H37Rv and MDR– MTBa |  | |
| 129e |  |  | MIC :  0.39 and 0.50 μg/mL | anti‐mycobacterial activity  MTB H37Rv and MDR– MTBa |  | |
| 129f |  |  | MIC:  0.78 and 0.25 μg/mL | anti‐mycobacterial activity  MTB H37Rv and MDR– MTBa |  | |
| 129g |  |  | MIC:  0.78 and 0.25 μg/mL | anti‐mycobacterial activity  MTB H37Rv and MDR– MTBa |  | |
| 129h |  |  | MIC:  0.78 and 0.25 μg/mL | anti‐mycobacterial activity  MTB H37Rv and MDR– MTBa |  | |
| 129i |  |  | MIC :  0.39 and 0.12 μg/mL | anti‐mycobacterial activity  MTB H37Rv and MDR– MTBa |  | |
| 129j |  |  | MIC:  0.20 and 0.12 μg/mL | anti‐mycobacterial activity  MTB H37Rv and MDR– MTBa |  | |
| 129k |  |  | MIC :  0.78 and 0.5 μg/mL | anti‐mycobacterial activity  MTB H37Rv and MDR– MTBa |  | |
| 129l |  |  | MIC :  0.39 and 0.25 μg/mL | anti‐mycobacterial activity  MTB H37Rv and MDR– MTBa |  | |
| 130 | Gatifloxacin‐1,2,3‐triazole‐isatin hybrids |  | MIC :  0.25 μg/mL | anti‐mycobacterial activity  MTB H37Rv | [63] | |
| 130a |  |  | MIC :  1 μg/mL | anti‐mycobacterial activity  MTB H37Rv |  | |
| 130b |  |  | MIC :  0.25 μg/mL | anti‐mycobacterial activity  MTB H37Rv |  | |
| 130c |  |  | MIC :  2 μg/mL | anti‐mycobacterial activity  MTB H37Rv |  | |
| 130d |  |  | MIC :  0.5 μg/mL | anti‐mycobacterial activity  MTB H37Rv |  | |
| 130e |  |  | MIC:  1 μg/mL | anti‐mycobacterial activity  MTB H37Rv |  | |
| 130f |  |  | MIC :  0.5 μg/mL | anti‐mycobacterial activity  MTB H37Rv |  | |
| 130g |  |  | MIC :  4 μg/mL | anti‐mycobacterial activity  MTB H37Rv |  | |
| 130h |  |  | MIC :  0.25 μg/mL | anti‐mycobacterial activity  MTB H37Rv |  | |
| 130i |  |  | MIC :  0.5 μg/mL | anti‐mycobacterial activity  MTB H37Rv |  | |
| 130j |  |  | MIC :  1 μg/mL | anti‐mycobacterial activity  MTB H37Rv |  | |
| 130k |  |  | MIC :  0.5 μg/mL | anti‐mycobacterial activity  MTB H37Rv |  | |
| 130l |  |  | MIC :  0.25 μg/mL | anti‐mycobacterial activity  MTB H37Rv |  | |
| 131 | Isatin-sulfonamide hybrids |  | Ki (caspace-3) = 60 nM  Ki (caspace-7) = 170 nM | Apoptosis  inhibit  caspase-1 caspase-3 caspase-6 caspase-7 | [43] | |
| 132 | Isatin-sulfonamide hybrids |  | Ki (caspace-3) = 15 nM  Ki (caspace-7) = 47 nM | Apoptosis  inhibit  caspase-1 caspase-3 caspase-6 caspase-7 | [43] | |
| 133 | Isatin-sulfonamide hybrids |  | Ki (caspace-3) = 436 nM  Ki (caspace-7) = 93 nM | Apoptosis  inhibit  caspase-1 caspase-3 caspase-6 caspase-7 | [43] | |
| 134 | Isatin-sulfonamide hybrids |  | Ki (caspace-3) = 42 nM  Ki (caspace-7) = 37 nM | Apoptosis  inhibit  caspase-1 caspase-3 caspase-6 caspase-7 | [43] | |
| 135 | Substituted isatin derivatives |  |  |  | [132] | |
| 135a |  |  | >10 µM | Anti-tumor activity (human tumor cell Jurkat) |  | |
| 135b |  |  | >10 µM | Anti-tumor activity (human tumor cell Jurkat) |  | |
| 135c |  |  | >10 µM | Anti-tumor activity (human tumor cell Jurkat) |  | |
| 135d |  |  | >10 µM | Anti-tumor activity (human tumor cell Jurkat) |  | |
| 135e |  |  | >10 µM | Anti-tumor activity (human tumor cell Jurkat) |  | |
| 135f |  |  | >10 µM | Anti-tumor activity (human tumor cell Jurkat) |  | |
| 135g |  |  | >10 µM | Anti-tumor activity (human tumor cell Jurkat) |  | |
| 135h |  |  | 0.20± 0.06 µM | Anti-tumor activity (human tumor cell Jurkat) |  | |
| 136 | Isatin–benzothiazole hybrid |  |  |  | [64] | |
| 136a |  |  | GI_50_ :  65.63 ± 1.81 µM | Anticancer (MDA-MB231 cancer cells) |  | |
| 136b |  |  | GI_50_:  60.61 ± 1.53 µM | Anticancer (MDA-MB231 cancer cells) |  | |
| 136c |  |  | G_I50_:  24.82 ± 0.75 µM | Anticancer (MDA-MB231 cancer cells) |  | |
| 136d |  |  | G_I50_:  38.03 ± 0.84 µM | Anticancer (MDA-MB231 cancer cells) |  | |
| 137 | Triazolyl-isatin hybrids |  | 140 ± 2 mM | Anti-inflammatory (inhibitory activity of TNF-a induced expression of Intercellular Adhesion Molecule-1 (ICAM-1) | [28] | |
| 138 | Triazolyl-isatin hybrids |  | 90 ± 1 mM | Anti-inflammatory(inhibitory activity of TNF-a induced expression of Intercellular Adhesion Molecule-1 (ICAM-1) | [28] | |
| 139 | Benzothiazole/Isatin-1,2,3-triazole-sulfa hybrids |  |  |  | [4] | |
| 139a |  |  | 175.62 ± 0.88 nM | Antitumor (inhibitory EGFR wild type assay) |  | |
| 139b |  |  | 290.30 ± 0.82 nM | Antitumor (inhibitory EGFR wild type assay) |  | |
| 139c |  |  | 424.20 ± 1.20 nM | Antitumor (inhibitory EGFR wild type assay) |  | |
| 139d |  |  | 668.97 ± 1.25 nM | Antitumor (inhibitory EGFR wild type assay) |  | |
| 139e |  |  | 468.21 ± 0.82 nM | Antitumor (inhibitory EGFR wild type assay) |  | |
| 140 | 1H-1,2,3-triazole-tethered isatin-7-chloroquinoline Hybrids |  |  |  | [27] | |
| 140a |  |  | 1.12 µM | Antiplasmodial activity (W2 (CQ-R) |  | |
| 140b |  |  | 1.17 µM | Antiplasmodial activity (W2 (CQ-R) |  | |
| 140c |  |  | 0.27 µM | Antiplasmodial activity (W2 (CQ-R) |  | |
| 140d |  |  | 0.81 µM | Antiplasmodial activity (W2 (CQ-R) |  | |
| 141 | 1H-1,2,3-triazole-tethered isatin-7-chloroquinoline Hybrids |  |  |  | [27] | |
| 141a |  |  | 1.01 µM | Antiplasmodial activity (W2 (CQ-R) |  | |
| 141b |  |  | 0.58 µM | Antiplasmodial activity (W2 (CQ-R) |  | |
| 141c |  |  | 0.42 µM | Antiplasmodial activity (W2 (CQ-R) |  | |
| 141d |  |  | 1.12 µM | Antiplasmodial activity (W2 (CQ-R) |  | |
| 142 | 1H-1,2,3-triazole linked b-lactameisatin bi-functional hybrids |  |  |  | [133] | |
| 142a |  |  | 44.51 mM | Antiparasitic  (*Trichomonas vaginalis*) |  | |
| 142b |  |  | 8.68 mM | Antiparasitic  (*Trichomonas vaginalis*) |  | |
| 142c |  |  | 7.06 mM | Antiparasitic  (Trichomonas vaginalis) |  | |
| 142d |  |  | 9.82 mM | Antiparasitic  (*Trichomonas vaginalis*) |  | |
| 142e |  |  | 7.69 mM | Antiparasitic  (Trichomonas vaginalis) |  | |
| 142f |  |  | 10.05 mM | Antiparasitic  (*Trichomonas vaginalis*) |  | |
| 142g |  |  | 22.09 mM | Antiparasitic  (*Trichomonas vaginalis*) |  | |
| 143 | triazole linked 3-benzylidene isatin hybrid |  | 3.7 ± 0.05 mM | Anticancer (DU145 (prostate) cells | [134] | |
| 144 | isatin oxadiazole hybrids |  | 7.80 ± 0.20 µM | Anticancer  (thymidine phosphorylase inhibitor) | [65] | |
| 145 | isatin oxadiazole hybrids |  | 9.40 ± 0.20 µM | Anticancer  (thymidine phosphorylase inhibitor) | [65] | |
| 146 | isatin oxadiazole hybrids |  | 5.30 ± 0.10 µM | Anticancer  (thymidine phosphorylase inhibitor) | [65] | |
| 147 | isatin oxadiazole hybrids |  | 16.50 ± 0.30 µM | Anticancer  (thymidine phosphorylase inhibitor) | [65] | |
| 148 | isatin oxadiazole hybrids |  | 19.40 ± 0.40 µM | Anticancer  (thymidine phosphorylase inhibitor) | [65] | |
| 149 | isatin oxadiazole hybrids |  | 6.20 ± 0.10 µM | Anticancer  (thymidine phosphorylase inhibitor) | [65] | |
| 150 | isatin oxadiazole hybrids |  | 5.30 ± 0.10 mM | Anticancer  (thymidine phosphorylase inhibitor) | [65] | |
| 151 | isatin oxadiazole hybrids |  | 4.70 ± 0.10 mM | Anticancer  (thymidine phosphorylase inhibitor) | [65] | |
| 152 | isatin oxadiazole hybrids |  | 17.60 ± 0.40 mM | Anticancer  (thymidine phosphorylase inhibitor) | [65] | |
| 153 | isatin oxadiazole hybrids |  | 48.50 ± 1.20 µM | Anticancer  (thymidine phosphorylase inhibitor) | [65] | |
| 154 | isatin oxadiazole hybrids |  | 38.60 ± 0.90 µM | Anticancer (thymidine phosphorylase inhibitor) | [65] | |
| 155 | isatin oxadiazole hybrids |  | 29.10 ± 0.90 µM | Anticancer  (thymidine phosphorylase inhibitor) | [65] | |
| 156 | isatin oxadiazole hybrids |  | 46.20 ± 1.20 µM | Anticancer  (thymidine phosphorylase inhibitor) | [65] | |
| 157 | isatin oxadiazole hybrids |  | 49.40 ± 1.30 µM | Anticancer  (thymidine phosphorylase inhibitor) | [65] | |
| 158 | isatin oxadiazole hybrids |  | 34.30 ± 0.60 µM | Anticancer  (thymidine phosphorylase inhibitor) | [65] | |
| 159 | isatin oxadiazole hybrids |  | 22.20 ± 0.60 µM | Anticancer  (thymidine phosphorylase inhibitor) | [65] | |
| 160 | isatin oxadiazole hybrids |  | 26.40 ± 0.60 µM | Anticancer  (thymidine phosphorylase inhibitor) | [65] | |
| 161 | isatin oxadiazole hybrids |  | 34.60 ± 0.70 µM | Anticancer  (thymidine phosphorylase inhibitor) | [65] | |
| 162 | isatin oxadiazole hybrids |  | 16.40 ± 0.40 µM | Anticancer  (thymidine phosphorylase inhibitor) | [65] | |
| 163 | isatin oxadiazole hybrids |  | 37.30 ± 0.80 µM | Anticancer  (thymidine phosphorylase inhibitor) | [65] | |
| 164 | isatin oxadiazole hybrids |  | 30.10 ± 0.60 µM | Anticancer  (thymidine phosphorylase inhibitor) | [65] | |
| 165 | isatin oxadiazole hybrids |  | 18.40 ± 0.40 µM | Anticancer  (thymidine phosphorylase inhibitor) | [65] | |
| 166 | isatin oxadiazole hybrids |  | 18.70 ± 0.40 µM | Anticancer  (thymidine phosphorylase inhibitor) | [65] | |
| 167 | isatin oxadiazole hybrids |  | 17.90 ± 0.50 µM | Anticancer  (thymidine phosphorylase inhibitor) | [65] | |
| 168 | isatin oxadiazole hybrids |  | 26.80 ± 0.60 µM | Anticancer  (thymidine phosphorylase inhibitor) | [65] | |
| 169 | Isatin-pyrazole benzenesulfonamide hybrids |  |  | Anticancer (human carbonic anhydrase isoforms hCA I, II, IX and XII) | [66] | |
| 169a |  |  | **Ki:** 53.7,11.8, 8.8, and 91.5 Nm |  |  | |
| 169b |  |  | **Ki:** 102, 9.9, 7.4 and 65.9 nM |  |  | |
| 169c |  |  | **Ki:** 9.0, 6.4, 20.0 and 83.6 nM |  |  | |
| 169d |  |  | **Ki:** 52.4 ,5.9 ,4.7 and 244 nM |  |  | |
| 170 | Isatin-pyrazole benzenesulfonamide hybrids |  |  | Anticancer (human carbonic anhydrase isoforms hCA I, II, IX and XII) | [66] | |
| 170a |  |  | **Ki:** 9.7, 4.3, 52.9 and 73.5 nM | Anticancer (human carbonic anhydrase isoforms hCA I, II, IX and XII) |  | |
| 170b |  |  | **Ki:** 38.3, 4.6, 9.7 and 44.5 nM | Anticancer (human carbonic anhydrase isoforms hCA I, II, IX and XII) |  | |
| 170c |  |  | **Ki:**6.7,5.5, 7.8 and 91.4 nM | Anticancer (human carbonic anhydrase isoforms hCA I, II, IX and XII) |  | |
| 170d |  |  | **Ki:** 7.6,3.5, 3.3 and 74.6 nM | Anticancer (human carbonic anhydrase isoforms hCA I, II, IX and XII) |  | |
| 170e |  |  | **Ki:** 49.5, 31.3, 15.7 and 3.7 nM | Anticancer (human carbonic anhydrase isoforms hCA I, II, IX and XII) |  | |
| 170f |  |  | **Ki:** 61.9, 17.9, 13.6 and 6.5 nM | Anticancer (human carbonic anhydrase isoforms hCA I, II, IX and XII) |  | |
| 171 | Isatin-pyrazole benzenesulfonamide hybrids |  |  |  | [66] | |
| 171a |  |  | **Ki:** 5.7, 2.9, 2.8 and 37.7 nM | Anticancer (human carbonic anhydrase isoforms hCA I, II, IX and XII) |  | |
| 171b |  |  | **Ki:** 7.1, 3.8,2.5 and 22.8 nM | Anticancer (human carbonic anhydrase isoforms hCA I, II, IX and XII) |  | |
| 171c |  |  | **Ki:** 5.2, 3.2, 9.4 and 56.8 nM | Anticancer (human carbonic anhydrase isoforms hCA I, II, IX and XII) |  | |
| 171d |  |  | **Ki:** 7.1, 4.5, 3.5 and 82.8 nM | Anticancer (human carbonic anhydrase isoforms hCA I, II, IX and XII) |  | |
| 172 | Benzofuran-isatin hybrids |  |  |  | [18] | |
| 172a |  |  | MIC:  4 and 4 µg/mL | Anti-mycobacterial activity  (MDR-MTB1 and MDR-MTB2) |  | |
| 172b |  |  | MIC : 8 and 4 µg/mL | Anti-mycobacterial activity  (MDR-MTB1 and MDR-MTB2 |  | |
| 172c |  |  | MIC:  2 and 2 µg/mL | Anti-mycobacterial activity  (MDR-MTB1 and MDR-MTB2) |  | |
| 172d |  |  | MIC:  4 and 2 µg/mL | Anti-mycobacterial activity  (MDR-MTB1 and MDR-MTB2) |  | |
| 172e |  |  | MIC:  4 and 8 µg/mL | Anti-mycobacterial activity  (MDR-MTB1 and MDR-MTB2) |  | |
| 172f |  |  | MIC:  1 and 2 µg/mL | Anti-mycobacterial activity  (MDR-MTB1 and MDR-MTB2) |  | |
| 172g |  |  | MIC:  8 and 16 µg/mL | Anti-mycobacterial activity  (MDR-MTB1 and MDR-MTB2) |  | |
| 173 | Benzofuran-isatin hybrids |  |  |  | [18] | |
| 173a |  |  | MIC:  8 and 16 µg/mL | Anti-mycobacterial activity  (MDR-MTB1 and MDR-MTB2) |  | |
| 173b |  |  | MIC:  8 and 4 µg/mL | Anti-mycobacterial activity  (MDR-MTB1 and MDR-MTB2) |  | |
| 173c |  |  | MIC:  16 and 16 µg/mL | Anti-mycobacterial activity  (MDR-MTB1 and MDR-MTB2) |  | |
| 174 | moxifloxacin-acetyl-1,2,3-1H-triazole-methylene isatin hybrids |  |  |  | [19] | |
| 174a |  |  | 4.0 µg/mL | anti-mycobacterial activity (MTB H_37_Rv) |  | |
| 174b |  |  | 6.0 µg/mL | anti-mycobacterial activity (MTB H_37_Rv) |  | |
| 174c |  |  | 5.0 µg/mL | anti-mycobacterial activity (MTB H_37_Rv) |  | |
| 174d |  |  | 8.0 µg/mL | anti-mycobacterial activity (MTB H_37_Rv) |  | |
| 175 | Benzofuran-isatin-imine hybrids |  |  |  | [39] | |
| 175a |  |  | MIC:  0.25 µg/mL | antibacterial activity (MSSE) |  | |
| 175b |  |  | MIC:  0.50 µg/mL | antibacterial activity (MSSE) |  | |
| 175c |  |  | MIC:  1 µg/mL | antibacterial activity (MSSE) |  | |
| 175d |  |  | MIC:  0.5 µg/mL | antibacterial activity Gram-positive strains (MSSE) |  | |
| 176 | isatin-Schiff’s base and chalcone hybrids |  | 11.40± 0.89, 4.73± 0.34 and 5.80± 0.45 μM/mL | anti-proliferative activities(MCF-7, HepG-2, and HCT-116) | [49] | |
| 177 | isatin-Schiff’s base and chalcone hybrids |  | 0.03, 2. 0.11 and 2.48± 0.12 μM/mL | anti-proliferative activities(MCF-7, HepG-2, and HCT-116) | [49] | |
| 178 | isatin-Schiff’s base and chalcone hybrids |  | 35.60± 1.6 ,18.60± 1.25 and 21.70± 1.4 μM/mL | anti-proliferative activities(MCF-7, HepG-2, and HCT-116) | [49] | |
| 179 | thiazolidinone-isatin hybrids |  |  |  | [67] | |
| 179a |  |  | 108.3 ± 8.2 μM | Anti-proliferative (MDA-MB-231 cell lines) |  | |
| 179b |  |  | 24.1 ± 2.1 μM | Anti-proliferative (MDA-MB-231 cell lines) |  | |
| 179c |  |  | 15.7 ± 0.9 μM | Anti-proliferative (MDA-MB-231 cell lines) |  | |
| 179d |  |  | 42.6 ± 3.4 μM | Anti-proliferative (MDA-MB-231 cell lines) |  | |
| 179e |  |  | 60.9 ± 4.2 μM | Anti-proliferative (MDA-MB-231 cell lines) |  | |
| 180 | thiazolo[3,2-*a*]benzimidazolone-isatin hybrids |  |  |  | [67] | |
| 180a |  |  | 47.2 *±* 3.8  μM | Anti-proliferative  (MDA-MB-231 cell lines) |  | |
| 180b |  |  | 13.2 *±* 1.7 μM | Anti-proliferative (MDA-MB-231 cell lines) |  | |
| 180c |  |  | 40.3 *±* 2.5 μM | Anti-proliferative (MDA-MB-231 cell lines) |  | |
| 181 | Isatin-thiazolo[3,2-a]benzimidazole hybrids |  |  |  | [68] | |
| 181a |  |  | 6.50 ± 0.32 μM | Anti-proliferative activity  (breast MDA-MB-231 cancer cell lines) |  | |
| 181b |  |  | 8.62 ± 0.51 μM | Anti-proliferative activity  (breast MDA-MB-231 cancer cell lines) |  | |
| 181c |  |  | 8.11 ± 0.33 μM | Anti-proliferative activity  (breast MDA-MB-231 cancer cell lines) |  | |
| 182 |  |  |  |  | [68] | |
| 182a |  |  | 2.60 ± 1.47 μM | Anti-proliferative activity  (breast MDA-MB-231 cancer cell lines) |  | |
| 182b |  |  | 5.45 ± 0.28 μM | Anti-proliferative activity  (breast MDA-MB-231 cancer cell lines) |  | |
| 182c |  |  | 7.65 ± 0.26  μM | Anti-proliferative activity  (breast MDA-MB-231 cancer cell lines) |  | |
| 183 | Ciprofloxacin-Isatin hybrid |  | MIC: 1.39 nmol/L | Anti-tuberculer (MTB H_37_Rv ) | [17] | |
| 184 | Isatin-azole hybrids |  | MIC: 6.25 μg/mL | antimycobacterial activity | [17] | |
| 185 | Isatin-thiazole azetidinone hybrids |  | 0.39 μmol/L | antimycobacterial activity (MTB H_37_Rv) | [17] | |
| 186 | Isatin pyrimidine hybrids |  | MIC: 100 μg/mL | Antimycobacterial (MTB H_37_Rv) | [17] | |
| 187 | Isatin-Carbohydrazone hybrids |  | 246.86 µM | Anti-oxidant activity (inhibition of DPPH radical.) | [24] | |
| 188 | Isatin-Carbohydrazone hybrids |  | 361.66 µM | Anti-oxidant activity (inhibition of DPPH radical) | [24] | |
| 189 | Isatin-Carbohydrazone hybrids |  | 1916.32 µM | Anti-oxidant activity (inhibition of DPPH radical) | [24] | |
| 190 | Isatin-benzoxazolehybrids |  |  |  | [22] | |
| 190a |  |  | MIC: 10-100 μg/ml | Anti-bacteria  (*E. coli* and *S.typhi*, *B. subtilis*) |  | |
| 190b |  |  | MIC: 10-100 μg/ml | Anti-bacteria  (*E. coli* and *S.typhi*, *B. subtilis*) |  | |
| 190c |  |  | MIC: 10-100 μg/ml | Anti-bacteria  (*E. coli* and *S.typhi*, *B. subtilis*) |  | |
| 190d |  |  | MIC: 10-100 μg/ml | Anti-bacteria  (*E. coli* and *S.typhi*, *B. subtilis*) |  | |
| 191 | Isatin-sulfonamide hybrids |  |  |  | [135] | |
| 191a |  |  | **Ki:** 173.6 and 43.9nM | Anticancer  (Carbonic anhydrase inhibitor  **hCA IX hCA XII)** |  | |
| 191b |  |  | **Ki:** 32.3 and 9.1 nM | Anticancer  (Carbonic anhydrase inhibitor  **hCA IX hCA XII)** |  | |
| 191c |  |  | **Ki:** 31.5 and 15.3 nM | Anticancer  (Carbonic anhydrase inhibitor  **hCA IX hCA XII)** |  | |
| 191d |  |  | **Ki:** 101.2 and 40.2 nM | Anticancer  (Carbonic anhydrase inhibitor  **hCA IX hCA XII)** |  | |
| 191e |  |  | **Ki:** 196.9 and 48.7 nM | Anticancer  (Carbonic anhydrase inhibitor  **hCA IX hCA XII)** |  | |
| 191f |  |  | **Ki:** 264.5 and 24.7 nM | Anticancer  (Carbonic anhydrase inhibitor  **hCA IX hCA XII)** |  | |
| 191g |  |  | **Ki:** 23.8  and 9.0 nM | Anticancer  (Carbonic anhydrase inhibitor  **hCA IX hCA XII)** |  | |
| 191h |  |  | **Ki:** 715.6  and 53.9  nM | Anticancer  (Carbonic anhydrase inhibitor  **hCA IX hCA XII)** |  | |
| 192 | Isatin-sulfonamide hybrids |  |  |  | [135] | |
| 192a |  |  | **Ki:** 30.6  and 65.6  nM | Anticancer  (Carbonic anhydrase inhibitor  **hCA IX hCA XII)** |  | |
| 192b |  |  | **Ki:** 8.9  and 9.2  nM | Anticancer  (Carbonic anhydrase inhibitor  **hCA IX hCA XII)** |  | |
| 192c |  |  | **Ki:** 30.1  and 24.7  nM | Anticancer  (Carbonic anhydrase inhibitor  **hCA IX hCA XII)** |  | |
| 192d |  |  | **Ki:** 177.2  and 16.2  nM | Anticancer  (Carbonic anhydrase inhibitor  **hCA IX hCA XII)** |  | |
| 192e |  |  | **Ki:** 27.4  and 44.1  nM | Anticancer  (Carbonic anhydrase inhibitor  **hCA IX hCA XII)** |  | |
| 192f |  |  | **Ki:** 77.9  and 43.3  nM | Anticancer  (Carbonic anhydrase inhibitor  **hCA IX hCA XII)** |  | |
| 192g |  |  | **Ki:** 17.7  and 9.6 nM | Anticancer  (Carbonic anhydrase inhibitor  **hCA IX hCA XII)** |  | |
| 193 | Isatin-sulfonamide hybrids |  |  |  | [135] | |
| 193a |  |  | **Ki:** 25.2  and 70.4 nM | Anticancer  (Carbonic anhydrase inhibitor  **hCA IX hCA XII)** |  | |
| 193b |  |  | **Ki:**  148.8  and 53.1 nM | Anticancer  (Carbonic anhydrase inhibitor  **hCA IX hCA XII)** |  | |
| 193c |  |  | **Ki:** 81.2 and 78.1  nM | Anticancer  (Carbonic anhydrase inhibitor  **hCA IX hCA XII)** |  | |
| 194 | Isatin-furan hybrids |  |  |  | [136] | |
| 194a |  |  | MIC:  8 and 8 μg/mL | Antitubercular(MTB H_37_Rv and MDR-MTB) |  | |
| 194b |  |  | MIC:  16 and 16 μg/mL | Antitubercular(MTB H_37_Rv and MDR-MTB) |  | |
| 194c |  |  | MIC:  8 and 16 μg/mL | Antitubercular(MTB H_37_Rv and MDR-MTB) |  | |
| 194d |  |  | MIC:  16 and 8 μg/mL | Antitubercular(MTB H_37_Rv and MDR-MTB) |  | |
| 194e |  |  | MIC:  32 and 32 μg/mL | Antitubercular(MTB H_37_Rv and MDR-MTB) |  | |
| 194f |  |  | MIC:  16 and 32 μg/mL | Antitubercular(MTB H37Rv and MDR-MTB) |  | |
| 194g |  |  | MIC:  2 and 4 μg/mL | Antitubercular(MTB H37Rv and MDR-MTB) |  | |
| 194h |  |  | MIC:  4 and 8 μg/mL | Antitubercular(MTB H37Rv and MDR-MTB) |  | |
| 194i |  |  | MIC:  4 and 4 μg/mL | Antitubercular(MTB H37Rv and MDR-MTB) |  | |
| 194j |  |  | MIC:  8 and 8 μg/mL | Antitubercular(MTB H37Rv and MDR-MTB) |  | |
| 195 | Isatin-quinoline hybrid |  | 24.9 nM | Antimalaria | [26] | |
| 196 | Isatin-quinoline hybrid |  | 216.0 nM | Antimalaria | [26] | |
| 197 | Isatin-quinoline hybrid |  | GI: 90–96% at 6.25 mg/mL | Antitubercular MTB H_37_ Rv | [17] | |
| 198 | Nitroimidazole-Isatin |  | *>*100 μM | Anti-cancer(MCF-7) | [137] | |
| 199 | Nitroimidazole-Isatin |  | *>*100 μM | Anti-cancer(MCF-7) | [137] | |
| 200 | Nitroimidazole-Isatin |  | >100 μM | Anti-cancer (MCF-7) | [137] | |

**References are as in the main mauscript**
